# Iron-Gallic Acid Peptide Nanoparticles as a Versatile Platform for Cellular Delivery with Synergistic ROS Enhancement Effect

**DOI:** 10.3390/pharmaceutics15071789

**Published:** 2023-06-21

**Authors:** Faqian Shen, Yi Lin, Miriam Höhn, Xianjin Luo, Markus Döblinger, Ernst Wagner, Ulrich Lächelt

**Affiliations:** 1 Pharmaceutical Biotechnology, Department of Pharmacy, Center for NanoScience (CeNS), LMU Munich, 81377 Munich, Germany; 2 Department of Chemistry, LMU Munich, 81377 Munich, Germany; 3 Department of Pharmaceutical Sciences, University of Vienna, 1090 Vienna, Austria

**Keywords:** intracellular delivery, peptide therapeutics, coordination nanoparticles, ROS, tumor combination therapy

## Abstract

Cytosolic delivery of peptides is of great interest owing to their biological functions, which could be utilized for therapeutic applications. However, their susceptibility to enzymatic degradation and multiple cellular barriers generally hinders their clinical application. Integration into nanoparticles, which can enhance the stability and membrane permeability of bioactive peptides, is a promising strategy to overcome extracellular and intracellular obstacles. Herein, we present a versatile platform for the cellular delivery of various cargo peptides by integration into metallo-peptidic coordination nanoparticles. Both termini of cargo peptides were conjugated with gallic acid (GA) to assemble GA-modified peptides into nanostructures upon coordination of Fe(III). Initial pre-complexation of Fe(III) by poly-(vinylpolypyrrolidon) (PVP) as a template favored the formation of nanoparticles, which are able to deliver the peptides into cells efficiently. Iron–gallic acid peptide nanoparticles (IGPNs) are stable in water and are supposed to generate reactive oxygen species (ROS) from endogenous H_2_O_2_ in cells via the Fenton reaction. The strategy was successfully applied to an exemplary set of peptide sequences varying in length (1–7 amino acids) and charge (negative, neutral, positive). To confirm the capability of transporting bioactive cargos into cells, pro-apoptotic peptides were integrated into IGPNs, which demonstrated potent killing of human cervix carcinoma HeLa and murine neuroblastoma N2a cells at a 10 µM peptide concentration via the complementary mechanisms of peptide-triggered apoptosis and Fe(III)-mediated ROS generation. This study demonstrates the establishment of IGPNs as a novel and versatile platform for the assembly of peptides into nanoparticles, which can be used for cellular delivery of bioactive peptides combined with intrinsic ROS generation.

## 1. Introduction

The impact of peptides on modern pharmaceutical research has been significant and played a crucial role in advancing the fields of biological and pharmaceutical sciences [1,2,3]. From the physicochemical perspective, bioactive peptides fill the gap between small molecule drugs and large macromolecular biologics, which can mediate therapeutic effects by interference with cellular processes and interactions with target receptors [4]. Compared to proteins, peptides generally exhibit moderate molecular weights, but at the same time also feature structural diversity and conformational flexibility [5], which can be tuned to specific bio-interactions. It was demonstrated that these remarkable biomolecules are suitable to treat cancer, vascular diseases, and microbial infections [6]. Furthermore, their design flexibility and feasible modification enables generation of sequences with favorable solubility, target selectivity, low toxicity and immunogenicity, leading to safe and economic therapeutics [7,8]. Nevertheless, certain limitations hinder the broad clinical application of peptides, such as short half-life and susceptibility to enzymatic degradation. In addition, the membrane impermeability of polar, macromolecular peptides generally restricts their applicability to extracellular targets, such as cell surface receptors, ion channels, or secreted proteins [2,9]. To unravel the full potential of peptide drugs and extend the pharmacological space of ‘druggable’ targets toward the intracellular environment, diverse strategies were developed, such as peptide cyclization, variation of the sequence length and side chains, and conjugation to cell-penetrating peptides (CPPs) [10]. The most widely used approach is the utilization of CPPs, which can readily be achieved by integration into the peptide sequence but does not address the proteolytic sensitivity and represents an extensive chemical modification of the native peptide [11].

Considering economic synthesis and retention of activity, nanoparticles (NPs) have played an increasing role in the delivery of biomacromolecules into cells, including therapeutic peptides [12,13,14,15,16]. Several researchers have confirmed that encapsulation of peptides into NPs strongly improves proteolytic stability and cellular uptake [17,18]. However, in contrast to nucleic acids, which can be encapsulated by nanocarriers via electrostatic interactions almost quantitatively, the diverse physicochemical properties of peptides impede the development of generic strategies for facile, flexible, and efficient cargo loading. Consequently, the general lack of active loading mechanisms frequently leads to low encapsulation efficiencies and capacities. Therefore, finding suitable concepts for delivery of bioactive peptides with facile and high loading efficiency, for instance via self-assembly, is still a challenging task.

Recently, extensive research showed that iron-based metal-phenolic networks (MPNs) have great potential for drug delivery and cancer therapy [19,20,21,22,23]. The contained polyphenols provide interaction points for hydrogen and covalent bond formation, π–π stacking, hydrophobic interactions, and metal coordination [20,24]. The polyphenolic compound gallic acid (GA) features several favorable characteristics for biomedical applications, such as antioxidant, anti-inflammatory, antimicrobial, and antitumoral properties, and is suggested as a beneficial agent for the treatment of diabetes and cardiovascular diseases [25,26,27,28,29,30]. Importantly, molecules with catechol or galloyl groups have the ability to chelate iron(III) and form complexes [31,32]. Furthermore, iron contained in MPNs can contribute to anticancer effects by generating reactive oxygen species (ROS) via the iron-induced Fenton reaction [33]. ROS, including peroxide (O_2_^2−^), O_2_^•−^, hydroxyl radicals (HO^•^), and singlet oxygen (^1^O_2_), play crucial roles in cell signaling and homeostasis in various biological processes [34,35]. However, an excessive accumulation of ROS can result in oxidative damage and disrupt proper cellular metabolism [36]. Consequently, harnessing external sources to induce ROS production has emerged as a promising strategy for cancer treatment. Various techniques, such as photodynamic therapy, molecular drugs, and biochemical reactions, can be employed to generate ROS. One notable example is the Fenton reaction, which utilizes iron to convert hydrogen peroxide (H_2_O_2_) into free radicals [37,38]. Therefore, combination therapies using iron-based nanocarriers to transport antitumoral agents and induce ROS formation present highly promising approaches with potential synergistic effects against cancer.

**Figure 1:**
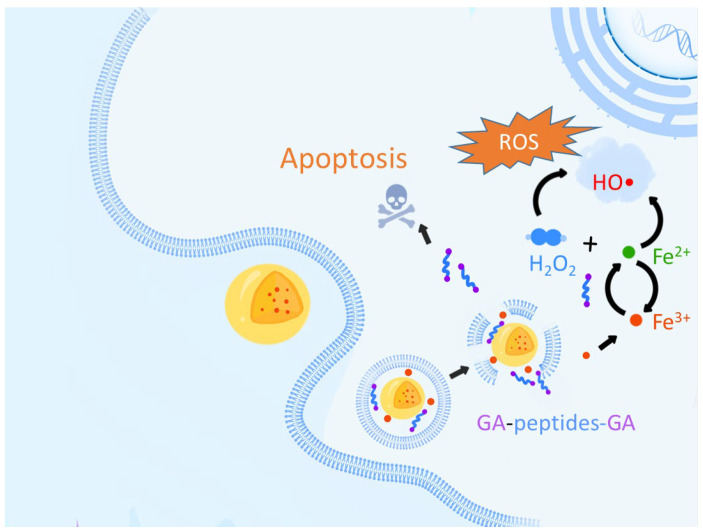
Illustration of combined antitumoral effects mediated by IGPNs containing gallic acid (GA)-modified apoptotic peptides and Fe^3+^.

Based on these considerations, we developed a novel generic platform for the quantitative assembly of peptides into nanoparticles and efficient cellular delivery. More specifically, a protected gallic acid (GA) derivative 3,4,5-tris((tert-butoxycarbonyl)oxy)benzoic acid [39] was used for the preparation of GA-functionalized peptides via solid-phase synthesis, which can assemble into iron–gallic acid peptide nanoparticles (IGPNs) via coordinative interaction with Fe^3+^. In view of potential applications in cancer therapy, pro-apoptotic peptides [40,41] were encapsulated into IGPNs to achieve multi-leveled antitumoral effects in combination with the intrinsic ROS generation mediated by the nanomaterial. Fe^2+^/Fe^3+^ can catalyze the conversion of endogenous H_2_O_2_ into highly reactive hydroxy radicals (HO^•^) through the Fenton reaction (Figure 1) [42,43]. In this process, polyphenols contribute by accelerating the reduction of Fe^3+^ to Fe^2+^ [44]. This strategy leads to therapeutic nanoparticles exclusively composed of functional components: bioactive peptides as well as ROS generating Fe^3+^ and gallic acid. In this work, the feasibility to assemble different peptides into IGPNs and trigger intracellular effects was systematically investigated. Key objectives of the study were to demonstrate the versatility of employing iron–gallic acid interactions for assembly of peptides into nanoparticles, to achieve high encapsulation efficiencies independent of the peptide sequence and confirm a preserved bioactivity. As a potential application, the presented platform provides a promising strategy for delivery of antitumoral peptides accompanied by synergistic chemodynamic effects via intracellular ROS generation.

## 2. Materials and Methods

Gallic acid (Sigma–Aldrich, Taufkirchen, Germany), anhydrous DCM AcroSeal^®^ (Acros Organics, Geel, Belgium), di-*tert*-butyl dicarbonate (DIBOC, Sigma–Aldrich), *N,N*-diisopropylethylamine (DIPEA, Iris Biotech), ethyl acetate (Sigma–Aldrich), 4-(dimethylamino)pyridine (DMAP, Sigma–Aldrich), 2-chlorotrityl chloride resin (Iris Biotech), Fmoc-L-Lys(Boc)-OH, Fmoc-L-Lys(Dde)-OH, Fmoc-L-Ala-OH, Fmoc-L-Thr(tBu)-OH, Fmoc-L-Gln(Trt)-OH, Fmoc-L-Pro-OH*H_2_O, Fmoc-L-Val-OH, Fmoc-L-Leu-OH, Fmoc-Gly-OH, Fmoc-L-Glu(tBu)-OH*H_2_O (Iris Biotech), 1-hydroxybenzotriazole (HOBt, Sigma–Aldrich), benzotriazole-1-yl-oxy-tris-pyrrolidino-phosphonium hexafluorophosphate (PyBOP, Multisyntech GmbH, Witten, Germany), methanol anhydrous AcroSeal^®^ (Acros Organics), *N,N*-dimethylformamide (DMF, Iris Biotech, Marktredwitz, Germany), dichloromethane (DCM, Bernd Kraft, Oberhausen, Germany), *N*-methyl-2-pyrrolidone (NMP, Iris Biotech), trifluoroacetic acid (TFA, Thermo Scientific, Waltham, MA, USA), piperidine (Iris Biotech), hydrazinium hydroxide (Sigma–Aldrich), methyl-*tert*-butyl ether (Brenntag Mülheim/Ruhr, Essen, Germany), *n*-hexane (Brenntag Mülheim/Ruhr), deuterium oxide (Sigma–Aldrich), dimethyl sulfoxide-*d₆* (Eurisotop, Saint-Aubin, France), iron (III) chloride hexahydrate (Grüssing GmbH, Filsum, Germany), polyvinylpyrrolidone (PVP, Sigma–Aldrich), 2′,7′-dichlordihydrofluorescein-diacetate (H_2_DCFDA, Thermo Fisher Scientific), CellROX™ Green Flow Cytometry Assay Kit (Thermo Fisher Scientific), CellTiter-Glo^®^ (Promega, Madison, WI, USA), methylene blue (Sigma–Aldrich).

### 2.1. Synthesis of 3,4,5-tri-O-(tert-butoxycarbonyl)-gallic Acid

The reagents 4-dimethylaminopyridine (DMAP) (61 mg, 0.5 mmol, 0.5 eq.), di-*tert*-butyl dicarbonate (DIBOC) (237 mg, 1.08 mmol, 4.0 eq.), and pyridine (474 mg, 6 mmol, 6.0 eq.) were added in sequence to a stirred suspension of gallic acid (170 mg, 1 mmol, 1.0 eq.) in dichloromethane (20 mL) under a nitrogen atmosphere. After stirring at room temperature overnight, the reaction mixture was quenched with water (20 mL) and washed with 3 × 1 M HCl, 3 × water, and dried over anhydrous MgSO_4_. The solvent was evaporated at reduced pressure to give the crude product. Purification by column chromatography with n-hexane:ethyl acetate 9:1 (*v*/*v*) as eluent yielded the product 3,4,5-tri-O-(tert-butoxycarbonyl)-gallic acid (231 mg, 47.1%) as a slightly yellow oil, ^1^H NMR (400 MHz, *DMSO-d_6_*) δ 7.78 (d, J = 0.6 Hz, 2H), 1.49 (s, 18H), 1.48 (s, 9H); MS (ESI) *m*/*z* 469.2 [(M-H)^−^].

### 2.2. General Synthesis of Peptides

A 2-chlorotrityl chloride resin was used as solid support for all peptide syntheses. In the case of GA modified peptides, Fmoc-Lys(Dde)-OH was loaded onto the peptide resin. Amino acids were sequentially coupled from the C- to N-terminus under standard Fmoc solid phase peptide synthesis (SPPS) conditions in 10 mL syringe reactors. Coupling of α-amino acids were carried out with 4 eq Fmoc L-amino acid (relative to loaded amines), 4 eq 1-hydroxybenzotriazole (HOBt), 4 eq PyBOP, and 8 eq *N,N*-diisopropylethylamine (DIPEA) dissolved in a mixture of DCM–DMF 4:3 (7 mL per g resin), followed by 1 h incubation under agitation at room temperature. Fmoc deprotection was carried out by 4 × 10 min incubation with 20% piperidine in DMF (7 mL per g resin) at room temperature. After each coupling and deprotection step, the resin was washed 2 × DMF and 3 × DCM (7 mL per g resin), and a Kaiser test [44] was performed to confirm quantitative conversion. In the case of double-GA modified peptides, the sequence was terminated by coupling of Fmoc-Lys(Dde)-OH, followed by Fmoc deprotection and Boc protection (10 eq DIBOC and 10 eq DIPEA in DCM, 1 h reaction time) of the lysine α-amine. Dde protecting groups were removed by 15 × 2 min incubation with 2% hydrazinium hydroxide in DMF (7 mL per g resin) at room temperature. Next, 3,4,5-tri-O-(*tert*-butoxycarbonyl)-gallic acid (Boc-protected GA) was coupled to the deprotected lysine ε-amines with a solution containing 3 eq of the building block, 3 eq PyBOP, 3 eq HOBt, and 12 eq DIPEA in DMF for 30 min at 50 °C (Biotage SP Wave) and for 30 min at room temperature. Finally, peptides were cleaved from the resin by incubation with trifluoroacetate–triisopropylsilane–H_2_O 95:2.5:2.5 (7 mL per g resin) for 90 min at room temperature. The cleavage solution was dropped into 45 mL of pre-cooled methyl-*tert*-butylether (MTBE)–*n*-hexane 1:1 and centrifuged. The supernatant was discarded, and the precipitated peptide was collected. Peptide purification was carried out by preparative RP–HPLC with a Pure C-830 chromatography system (BÜCHI, Flawil, Switzerland), a semipreparative C18 RP–HPLC column (Waters, Milford, USA), and a gradient from 99:1 to 0:100 (water/acetonitrile) within 25 min. All peptides were lyophilized and analyzed by analytic RP-HPLC, ^1^H-NMR, and mass spectrometry (MALDI–MS or ESI–MS).

### 2.3. Synthesis of Iron–Gallic Acid Peptide Nanoparticles (IGPNs)

For the synthesis of FePVP nanoparticles, a solution of FeCl_3_ · 6H_2_O in H_2_O (0.2 mL, 0.6 M) was added to 10 mL PVP solution (10 mg mL^−1^ in H_2_O) and agitated for 1 h. The peptide solution (1 mL, 0.6 M in H_2_O) was added to the mixture and stirred overnight. Fe nanoparticles were synthesized in a similar way, but without addition of PVP. The following day, the obtained nanoparticles were dialyzed (MWCO = 10 K) against deionized water for 24 h and stored at 4 °C for further use.

### 2.4. Investigation of the Stability of IGPNs in Different Media

In order to evaluate the stability of IGPNs over time, IGPNs were incubated in water, PBS, 50% serum, and artificial lysosomal fluid (ALF 1 L contains: 3.21 g NaCl, 6.00 g NaOH, 20.80 g citric acid, 0.097 g CaCl_2_, 0.179 g sodium phosphate heptahydrate, 0.039 g Na_2_SO_4_, 0.106 g MgSO_4_ × 6 H_2_O, 0.059 g glycerine, 0.077 g sodium citrate dihydrate, 0.09 g sodium tartrate dihydrate, 0.085 sodium lactate, 0.086 sodium pyruvate, 1 mL formaldehyde). A total of 50 μL of the freshly prepared IGPNs were added into each cuvette, followed by dilution with 2 mL of the individual medium (water, PBS, 50% serum, and ALF). The four samples were monitored under agitation for 6 h (25 °C, 700 rpm) at 550 nm using a Cary 3500 UV–Vis Spectrophotometer. The absorbance of each sample was recorded every 5 min.

### 2.5. Methylene Blue Assay

MB assays were used to measure the HO^•^ production efficacy of IGPNs. IGPNs (36 µg Fe^3+^) were mixed with MB (1.5 mL, 10 µg L^−1^) and H_2_O_2_ (1 mL), and H_2_O was added to a final volume of 3 mL. The absorbance at 664 nm was recorded with a Cary 3500 UV–Vis Spectrophotometer every 5 min over 6 h.

### 2.6. Cell Culture

HeLa and N2a cells were grown in Dulbecco’s Modified Eagle Medium (DMEM) low glucose (1 g/L glucose) supplemented with 10% fetal bovine serum (FBS), 100 U/mL penicillin, and 100 μg/mL streptomycin. The cells were cultured in ventilated flasks at 37 °C and 5% CO_2_ in an incubator with a relative humidity of 95%. The cells were passaged at a confluency of 80–90%.

### 2.7. Evaluation of ROS Generation by Flow Cytometry

The CellROX™ Green Flow Cytometry Assay Kit (Thermo Fisher Scientific) was used to detect ROS in living cells by flow cytometry. HeLa cells were seeded in 24-well plates at a density of 50,000 cells/well one day prior to the treatment. On the next day, the medium was replaced with 450 μL of fresh medium. A total of 50 μL of peptides or IGPNs (100 μM) was added to each well, resulting in a final concentration of 10 μM peptides, and the cells were incubated for 24 h. Afterwards, the medium was removed, and the cells were harvested, washed, and re-suspended in PBS containing 10% FBS. The CellROX™ Green reagent was added to each sample resulting in a final concentration of 800 nM. Then, the cells were incubated for 45 min at 37 °C in the dark. Subsequently, 0.6 μL of the 5 μM SYTOX^®^ Red Dead Cell stain solution was added to each sample. After another 15 min incubation, the cells were analyzed by flow cytometry, as described above. The CellROX™ Green signal was measured with 488 nm excitation and 530 nm emission. The SYTOX^®^ Red signal was assayed with 639 nm excitation and 660 nm emission. Ten thousand isolated live cells were counted.

### 2.8. Evaluation of ROS Generation by Confocal Laser Scanning Microscopy (CLSM)

HeLa cells were seeded in 8-well Ibidi μ-slides (Ibidi GmbH, Gräfelfing, Germany) at a density of 20,000 cells/well 24 h prior to the treatment. On the next day, the medium was replaced with 270 μL of fresh medium. A total of 30 μL of IGPNs and peptides was added to each well, resulting in a final concentration of 10 μM peptides, and the cells were incubated for 24 h. As a positive control, tert-butyl hydroperoxide solution (TBHP, 200 μM) was added, and the cells were incubated for 30 min. The cells were then washed twice with PBS followed by 30 min of staining with H_2_DCFDA (10 μM) in the dark. Next, the H_2_DCFDA solution was discarded, and 300 μL of PBS was added per well for CLSM imaging. Images were recorded on a Leica-TCS-SP8 confocal laser scanning microscope equipped with a HC PL APO 63× 1.4 objective (Germany). The H_2_DCFDA signal was recorded with emission at 520 nm. All images were analyzed using the LAS X software from Leica.

### 2.9. Evaluation of Cellular Uptake by Flow Cytometry

The peptides were labeled using 5-carboxyfluorescein NHS ester. Peptides (5 mg) were dissolved in 0.5 mL of HEPES buffer (pH 7.4) and the pH was adjusted to 8.3 using 1 M NaOH. A solution of 5-carboxyfluorescein NHS ester (10 mg/mL) in DMSO was prepared. The solution of reactive dye was mixed with the peptide solution with a molar ratio of 0.75 (dye):1 (peptide). After a 4 h reaction time at room temperature, the peptide-carboxyfluorescein conjugate was purified by dialysis (MWCO = 1 KDa) and lyophilized for further use.

One day prior to the cellular uptake experiments, HeLa cells were seeded into 24-well plates at a density of 30,000 cells/well. On the next day, the medium in each well was replaced with 450 μL of fresh medium. Peptides (20% carboxyfluorescein labeled) and IGPNs were prepared as described above. A total of 50 μL of the nanoparticles was added to each well, resulting in a final concentration of 10 μM peptides, and the cells were incubated for 4 h. Subsequently, the cells were harvested, washed with PBS, and re-suspended in PBS containing 10% FBS. Trypan blue (0.04%, *w*/*v*) was added to quench extracellular carboxyfluorescein fluorescence, and the cells were incubated for 1 min. Then, 1 ng/μL DAPI was added directly to each sample before the measurement to differentiate between live and dead cells. The samples were analyzed by flow cytometry with a CytoFLEX S flow cytometer (Beckman Coulter, Brea, CA, USA). The DAPI fluorescence was detected with 405 nm excitation and 450 nm emission. The carboxyfluorescein signal was measured with 488 nm excitation and 530 nm emission. Ten thousand isolated live cells were counted and evaluated. The data were analyzed using FlowJo 7.6.5 by FlowJo, LLC (Becton, Dickinson and Company, Franklin Lakes, NJ, USA). All experiments were performed in triplicates.

### 2.10. Evaluation of Cellular Uptake by Confocal Laser Scanning Microscopy (CLSM)

HeLa cells were seeded in 8-well Ibidi μ-slides (Ibidi GmbH, Germany) at a density of 20,000 cells/well 24 h prior to the experiment. On the next day, the medium was replaced with 270 μL of fresh medium. A total of 30 μL of carboxyfluorescein-labeled IGPNs was added to each well, corresponding to a final concentration of 10 μM peptides. The medium was removed after 4 h incubation, and the cells were washed twice with 300 μL of PBS followed by 40 min of fixation with 4% PFA at RT. The cells were then washed twice with PBS again, and the cell nuclei were stained with 2 ng/μL DAPI. The DAPI solution was discarded after 20 min incubation, and the cells were further washed with trypan blue (0.04%, *w*/*v*). Next, 300 μL of PBS was added per well for CLSM imaging. Images were recorded on a Leica-TCS-SP8 confocal laser scanning microscope equipped with a HC PL APO 63 × 1.4 objective (Germany). DAPI and carboxyfluorescein emission were recorded at 450 nm and 520 nm, respectively. All images were analyzed using the LAS X software from Leica.

### 2.11. Evaluation of Cell Viability (CellTiter-Glo Assay)

The CellTiter-Glo^®^ Luminescent Cell Viability Assay was performed to determine the viability of HeLa or N2a cells after treatment with IGPNs or the individual components. One day prior to the treatments, HeLa or N2a cells were seeded into 96-well plates at a density of 5000 cells/well. On the next day, the medium in each well was replaced with 90 μL of fresh medium, and 10 μL of nanoparticles or control solutions (free peptides, Fe, FePVP) was added to each well corresponding to the specified final concentrations of the peptide or Fe^3+^, respectively. The supernatant was removed after 72 h incubation, and 25 µL of medium as well as 25 µL of CellTiter-Glo reagent were added to each well. The plate was gently agitated for 30 min at RT, and the luminescence signal (relative light units, RLU) was recorded by a Centro LB 960 plate reader luminometer (Berthold Technologies, Bad Wildbad, Germany). The relative cell viability (in percentage) was calculated relative to control wells treated with HBG buffer as ([RLU] test/[RLU] control) × 100%. All experiments were performed in triplicate.

### 2.12. Apoptosis Assay

Cell apoptosis was assessed by flow cytometry using an Annexin V–FITC Apoptosis Detection Kit (BioVision, Milpitas, CA, USA). HeLa cells were seeded into 24-well plates at a density of 30,000 cells/well one day prior to the experiment. On the next day, the medium in each well was replaced with 450 μL of fresh medium. A total of 50 μL of IGPNs or controls was added to each well corresponding to a final concentration of 10 μM peptides (or Fe in case of FeCl_3_ and FePVP). After 48 h incubation, the cells were trypsinized, collected, and re-suspended in 500 μL of Annexin V–FITC binding buffer (1×). Next, 5 μL of Annexin V–FITC and 5 μL of propidium iodide (PI, 50 μg/mL) were added to each sample, and the samples were incubated for 5 min at RT in the dark. Afterwards, the cells were washed, re-suspended, and analyzed by flow cytometry as described above. The FITC signal was measured with 488 nm excitation and 530 nm emission. The PI fluorescence was assayed with 488 nm excitation and 640 nm emission. All experiments were performed in triplicate.

## 3. Results and Discussion

### 3.1. Synthesis of Gallic Acid-Tagged Peptides

**Scheme 1:**
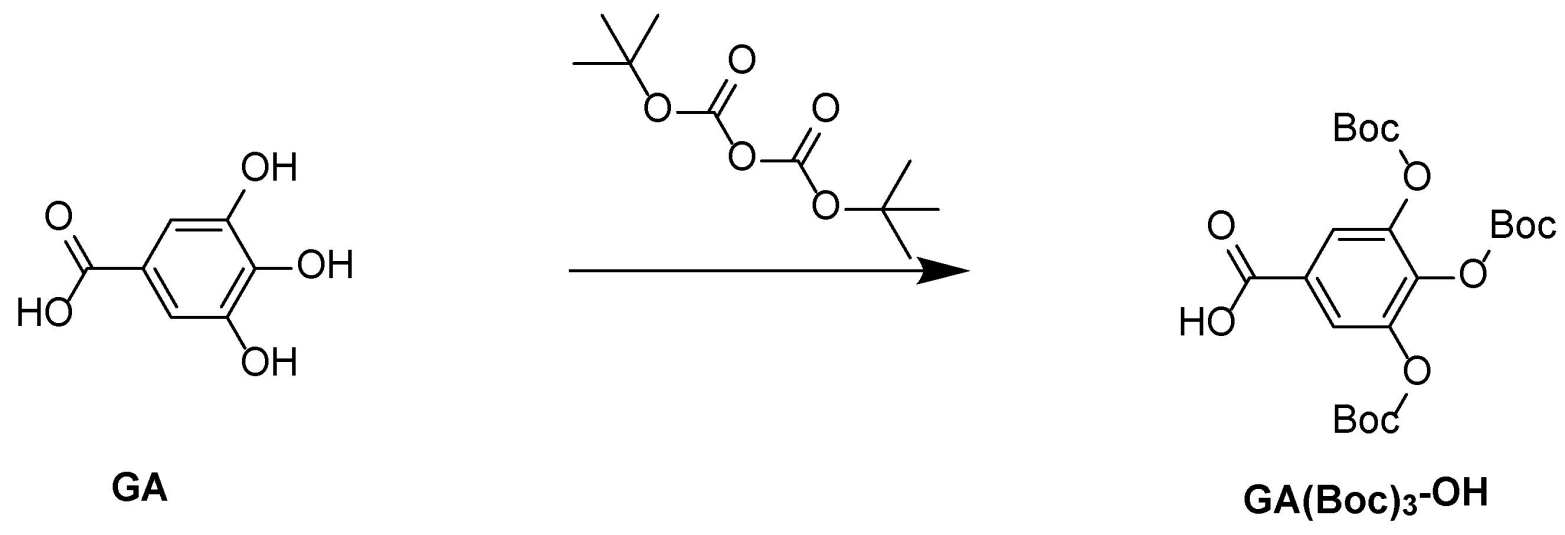
Synthesis of Boc-protected gallic acid. Reagents and conditions: DIBOC (4 eq), pyridine (6 eq), DMAP (0.5 eq), DCM, RT, 2 h.

The basis for the assembly of peptides into IGPNs is the integration of gallic acid (GA) into the peptide sequences. To enable GA functionalization during peptide synthesis, a Boc-protected derivative was synthesized following a procedure developed by Biessen et al. (Scheme 1): gallic acid was converted into 3,4,5-tri-O-(tert-butoxycarbonyl)-gallic acid (GA(Boc)_3_-OH) by reaction with di-tert-butyldicarbonate in presence of pyridine and 4-dimethylaminopyridine [39]

Standard Fmoc solid-phase peptide synthesis conditions were used for the assembly of a series of GA modified peptides (Table 1). The sequences were designed for a systematic assessment of the following peptide sequence parameters: (1) number of GA moieties, (2) physico–chemical peptide characteristics, and (3) bioactivity. The different peptides can be grouped into six series: minimal GA peptide motif, neutral model peptides, positively charged model peptides, negatively charged peptides, pro-apoptotic SIO peptides, and pro-apoptotic AVP peptides. Each sequence was conjugated with one or two gallic acid moieties via amide formation at the side chain of N- and C-terminal lysines serving as molecular adaptors. The purity, molecular weights, and structures of all synthesized peptides were confirmed by analytic reversed-phase high-performance liquid chromatography (RP–HPLC), electrospray ionization (ESI)- or matrix-assisted laser desorption/ionization time-of-flight (MALDI–TOF) mass spectrometry, and nuclear magnetic resonance spectroscopy (NMR) (cf. Supplementary Materials).

### 3.2. Synthesis and Characterization of Iron–Gallic Acid Nanoparticles (IGPNs)

**Figure 2:**
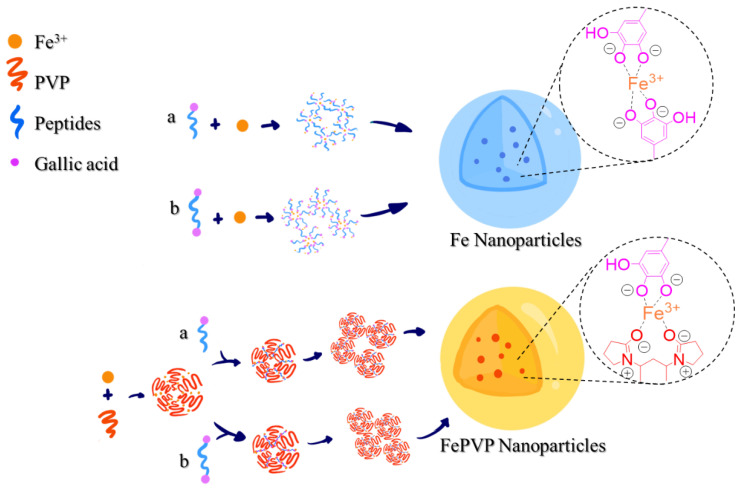
Synthesis of IGPNs by coordinative interaction of (a) mono- or (b) di-functionalized gallic acid peptides with Fe^3+^ (Fe nanoparticles), and with initial pre-complexation of Fe^3+^ with PVP (FePVP nanoparticles).

A previously published procedure for the generation of iron–GA coordination polymers was adapted to utilize the coordinative interaction between GA and Fe^3+^ [45,46] for the assembly of IGPNs. With each peptide, two different types of nanoparticles were assembled, either by direct interaction of GA modified peptides with Fe^3+^ in solution or after initial pre-complexation of Fe^3+^ with polyvinyl pyrrolidone (PVP) (Figure 2) [47]. FeCl_3_ was dissolved in bidistilled water for direct preparation of Fe nanoparticles, while for FePVP nanoparticles, FeCl_3_ and PVP were agitated for 1 h initially. Equimolar amounts of peptides were then added to assemble IGPNs via coordinative interactions between GA and Fe^3+^.

**Figure 3:**
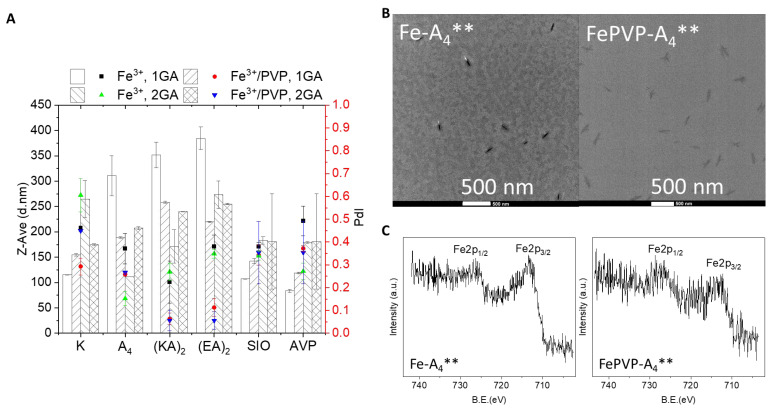
Characterization of nanoparticles. (**A**) Hydrodynamic size (z-average, columns) and polydispersity index (PdI, dots) of IGPNs as determined by DLS. (**B**) TEM micrograph of Fe-A_4_** and FePVP-A_4_**. (**C**) XPS analysis of Fe-A_4_** (**left**) and FePVP-A_4_** (**right**).

**Figure 4:**
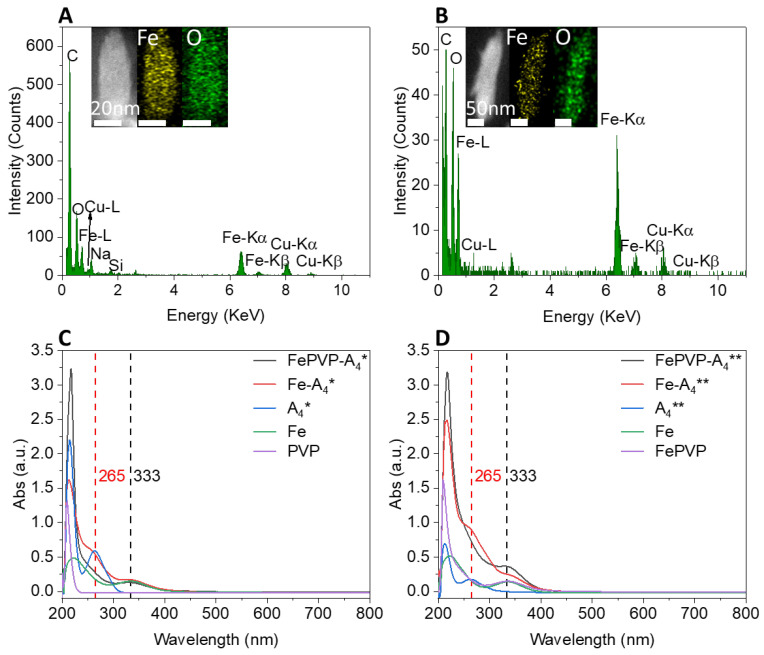
Molecular composition of IGPNs. (**A**) STEM-EDX elemental map of Fe-A_4_**. (**B**) STEM-EDX elemental map of FePVP-A_4_**. (**C**,**D**) UV–Vis spectra of FeCl_3_ (Fe), PVP, FePVP, A_4_*, Fe-A_4_*, FePVP-A_4_*, A_4_**, Fe-A_4_**, and FePVP-A_4_** after acidic decomposition of IGPNs.

The systematic evaluation showed that the formation of IGPNs depended on GA modification: native peptides without GA did not form particles with Fe^3+^, whereas peptides with one (*) or two (**) GA moieties at the termini showed assembly. Measurements of particles size distribution (hydrodynamic diameter), polydispersity index (PdI), and ζ- potential of the generated IGPN suspensions were carried out by dynamic and electrophoretic light scattering (DLS, ELS) (Figure 3A and Figure S2, Table S1) and revealed mean z-averages between 86.5 ± 2.7 nm and 384.4 ± 22.4 nm and ζ-potential values in the range between −25.2 mV and 2.2 mV. It was found that the length or charge of investigated peptide sequences, as well as the number of attached GA modifications, did not change the size significantly, However, in the case of positively or negatively charged peptide sequences, a strong reduction of PdI was achieved with the PVP-mediated synthesis procedure. Accordingly, an evaluation of the size distribution (Figure S2) suggests favorability of the PVP assembly approach due to a reduction of IGPN aggregation in an aqueous environment. In a dry, dispersed state, transmission electron microscopy (TEM) determined a similar appearance of Fe and FePVP nanoparticles built from neutral model peptides (Fe-A_4_** and FePVP-A_4_**, Figure 3B and Figure S3). X-ray diffraction (XRD) experiments with Fe-A_4_*, FePVP-A_4_*, Fe-A_4_**, and FePVP-A_4_** did not show distinct diffraction patterns (Figure S4). The XPS spectra of the iron 2p core level confirms the presence of iron in the sample. The core level for Fe(0) is expected at 707 eV. The binding energies shifted to higher values indicate higher oxidation states. Elemental mapping by energy dispersive X-ray (EDX) spectroscopy in scanning transmission electron microscopy (STEM) mode shows that iron (Fe) and oxygen (O) are homogeneously distributed in IGPNs (Figure 4A,B), and the total iron content in Fe-A_4_** and FePVP-A_4_** was determined by inductively coupled plasma optical emission spectroscopy (ICP–OES) to be ∼4.6% and ∼3.6%, respectively.

Furthermore, to assess the encapsulation efficiency of integrated peptides, solid IGPNs were separated from surrounding dispersant medium with spin filters (Agilent Spin Filter, 10 kD) via centrifugation (12,500 rpm, 5 min). Free peptide solutions (A_4_*, A_4_**, K*, K**, SIO*, SIO**, AVP*, AVP**) served as controls and analysis via RP–HPLC showed that the GA-modified peptides were encapsulated into IGPNs via Fe^3+^ chelation quantitatively (Figure S5), since in the case of IGPN suspensions, no distinct amounts of free peptide were detectable. The peptides were found to be quantitatively integrated in each of the four different IGPNs, providing strong evidence for an efficient assembly and encapsulation process.

**Figure 5:**
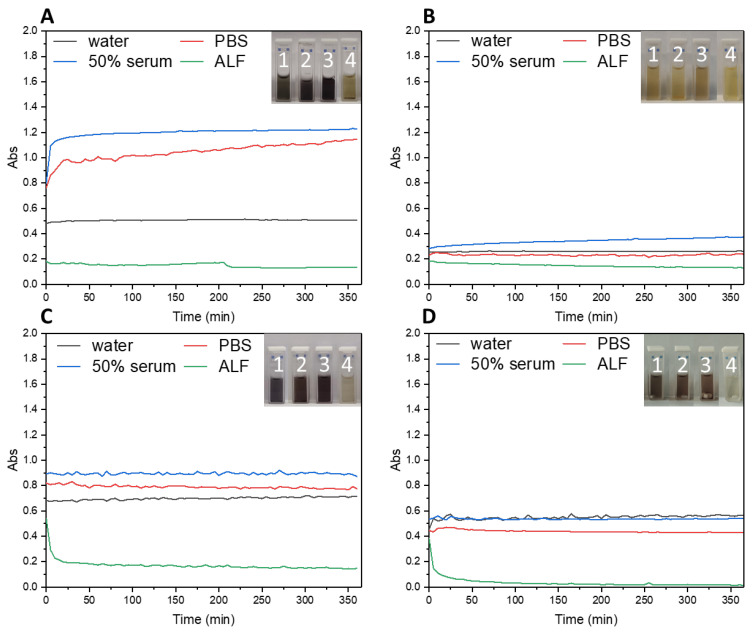
Absorbance of IGPNs at 550 nm in different media (1: water, 2: PBS, 3: 50% serum, 4: ALF). (**A**) Fe-A_4_*. (**B**) FePVP-A_4_*. (**C**) Fe-A_4_**. (**D**) FePVP-A_4_**.

Additional characterization of IGPNs was conducted by UV–Vis spectroscopy (Figure 4C,D and Figure S6). After decomposition of IGPNs at pH 1, the measurements revealed characteristic absorbance peaks of the particle components (265 nm peptide, 333 nm Fe^3+^). Compared with Fe nanoparticles (red curve), the FePVP nanoparticles (black curve) exhibited higher absorbance at 214 nm in relation to 265 nm, which can be attributed to the introduction of PVP. Moreover, the decomposition resulted in the disappearance of the Ligand to Metal Charge Transfer Bands (LMCT), which confirms the supposed nature of bonding between iron and gallic acid (Figure S7). Thermogravimetric analysis (TGA) showed an obvious difference between Fe-A_4_** and FePVP-A_4_** nanoparticles, which is consistent with a higher organic content due to the integration of PVP (Figure S8). The stability of Fe-A_4_*, FePVP-A_4_*, Fe-A_4_**, and FePVP-A_4_** in water, phosphate buffer (PBS), 50% serum, and artificial lysosomal fluid (ALF) were investigated by monitoring the absorbance of IGPN suspensions at 550 nm by UV–Vis spectroscopy (Figure 5).

ALF was prepared as previously described to simulate the environment of lysosomes with respect to the pH, ionic strength, salts, and viscosity [48]. The nanoparticles were assessed continuously over a time period of 6 h after the treatment with the abovementioned solutions. It was found that all IGPNs remained stable in water but dissolved rapidly in ALF. Interestingly, Fe-A_4_* and FePVP-A_4_* exposed to 50% serum increased in absorbance over time, presumably due to the adsorption of serum proteins onto the nanoparticle surface. In contrast, Fe-A_4_** and FePVP-A_4_** did not show this change of absorbance.

### 3.3. Cellular Uptake of IGPNs

**Figure 6:**
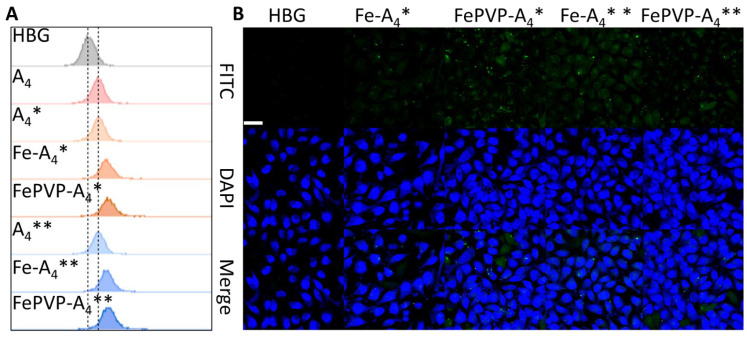
Cellular uptake of carboxyfluorescein-labeled peptides determined by flow cytometry and CLSM. (**A**) Flow cytometry analysis of HeLa cells treated with carboxyfluorescein-labeled A_4_ peptides or derived IGPNs for 4 h. (**B**) CLSM images of HeLa cells treated with HBG buffer, carboxyfluorescein-labeled Fe-A_4_*, FePVP-A_4_*, Fe-A_4_**, or FePVP-A_4_** for 4 h. Nuclei were stained with DAPI (blue) and FITC indicates carboxyfluorescein fluorescence (green). Additional flow cytometry and CLSM data are provided in Figures S9–S11. Scale bar, 40 μm.

The cellular uptake of IGPNs was investigated to confirm that the assembly into nanoparticles is a favorable strategy to facilitate cellular delivery of non-permeable peptides. Carboxyfluorescein-labeled derivatives of the model peptides with poor cell permeability (A_4_, A_4_*, A_4_**, (EA)_2_, (EA)_2_*, (EA)_2_**) were used for assembly into fluorescent IGPNs (Fe-A_4_*, FePVP-A_4_*, Fe-A_4_**, FePVP-A_4_**, Fe-(EA)_2_*, FePVP-(EA)_2_*, Fe-(EA)_2_**, FePVP-(EA)_2_**) and cellular internalization was determined by flow cytometry analysis (Figure 6A and Figure S9) and confocal laser scanning microscopy (CLSM, Figure 6B, Figures S10 and S11). As expected, in cells treated with free carboxyfluorescein-labeled peptides, no intense fluorescence enhancement was observed compared to HBG buffer-treated cells. In contrast, assembly into IGPNs strongly promoted cellular delivery of A_4_*, A_4_**, (EA)_2_*, and (EA)_2_**. Moreover, the CLSM images confirm that with IGPNs (Fe-A_4_*, FePVP-A_4_*, Fe-A_4_**, and FePVP-A_4_**), intracellular carboxyfluorescein fluorescence was detectable, in contrast to the free carboxyfluorescein-labeled peptides (A_4_, A_4_*, A_4_**) alone. Similar observations were made with the (EA)_2_ series. Altogether, the results demonstrate that IGPNs represent a feasible strategy to facilitate cellular uptake of non-permeable peptides.

### 3.4. ROS Production of IGPNs

**Figure 7:**
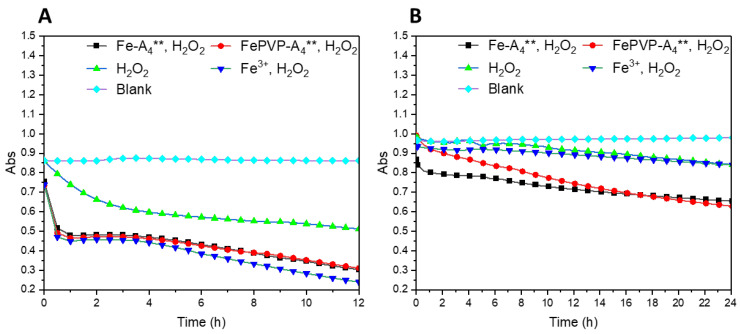
Determination of ROS generation by UV–Vis spectrometry. (**A**,**B**) Detection of HO^•^ at pH 4 (**A**) and pH 7.4 (**B**) by monitoring of methylene blue absorbance at 664 nm.

To verify the hypothesized Fenton reaction of IGPNs, H_2_O_2_ was used as a source of hydroxyl radicals (HO^•^) and to simulate the tumor microenvironment. The generation of HO^•^ and degradation of methylene blue (MB) upon exposure to H_2_O_2_ and different samples adjusted to the same Fe content (IGPNs or FeCl_3_ solution) was monitored by UV–Vis spectrometry [37]. At pH 4 (Figure 7A), the decrease of absorbance at 664 nm was accelerated in the presence of Fe-A_4_**, FePVP-A_4_**, or Fe^3+^. At the favorable acidic pH, no differences in reaction rates were observed between IGPNs and Fe^3+^, since the content of Fe^3+^ is obviously sufficient to drive the reaction under these conditions. In contrast, at pH 7.4, the absorbance decline was clearly more pronounced in case of IGPNs (Figure 7B). This phenomenon could be explained by an accelerated conversion of Fe^3+^ to Fe^2+^ in the presence of GA. The results indicate that IGPNs, aided by GA, facilitate the generation of toxic ROS though the Fenton reaction, which is expected to enhance their therapeutic effect.

**Figure 8:**
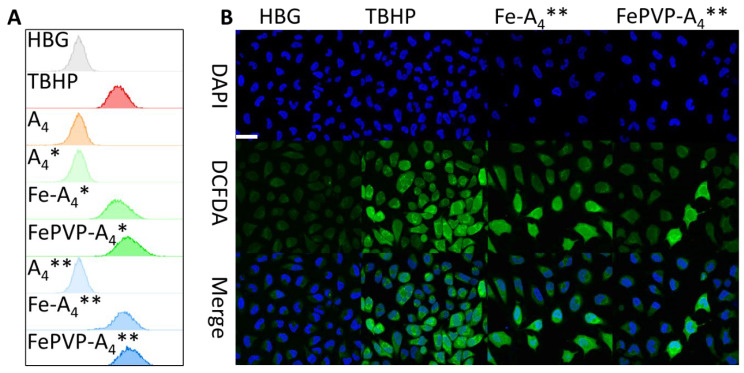
Determination of intracellular ROS generation by flow cytometry and confocal microscopy. (**A**) HeLa cells incubated with A_4_ peptides and derived IPGNs (10 μM, 24 h) followed by staining with CellROX Green. (**B**) HeLa cells incubated with Fe-A_4_** and FePVP-A_4_** (10 μM, 24 h) followed by staining with DCFDA. TBHP was used as a positive control. Scale bar, 40 μm.

To further evaluate ROS production on a cellular level, flow cytometry and confocal laser scanning microscopy (CLSM) were employed. CellROX™ Green reagent, which is converted to a bright fluorescent derivative intracellularly in presence of ROS, was incubated with HeLa cells pretreated with A_4_*, Fe-A_4_*, FePVP-A_4_*, A_4_**, Fe-A_4_**, and FePVP-A_4_**. HBG and tert-butyl hydroperoxide (TBHP) treated cells served as negative and positive controls, respectively. After 45 min of incubation, dead cells were stained with SYTOX red. After the evaluation of 10,000 live cells by flow cytometry, it was found that IGPNs (Fe-A_4_*, FePVP-A_4_**, Fe-A_4_**, and FePVP-A_4_**) mediated a distinct shift of the cell populations towards higher CellROX™ Green fluorescence, comparable to the positive control TBHP (Figure 8A). A visual confirmation of intracellular ROS generation was achieved by CLSM using the ROS imaging probe 2′,7′-dichlorodihydrofluorescein diacetate (DCFDA), which is oxidized to 2′,7′-dichlorofluorescein (DCF) upon intracellular exposure to ROS. Compared with the HBG group, a much stronger green fluorescence could be observed in cells after 24 h of incubation with IGPNs (Figure 8B). Additional data indicates that the IPGN-triggered ROS generation is time and dose dependent (Figure S12). In summary, the IPGNs (Fe-A4** and FePVP-A4**) exhibited a notable capacity to induce the production of ROS within HeLa cells.

### 3.5. Bioactivity of IGPNs

**Figure 9:**
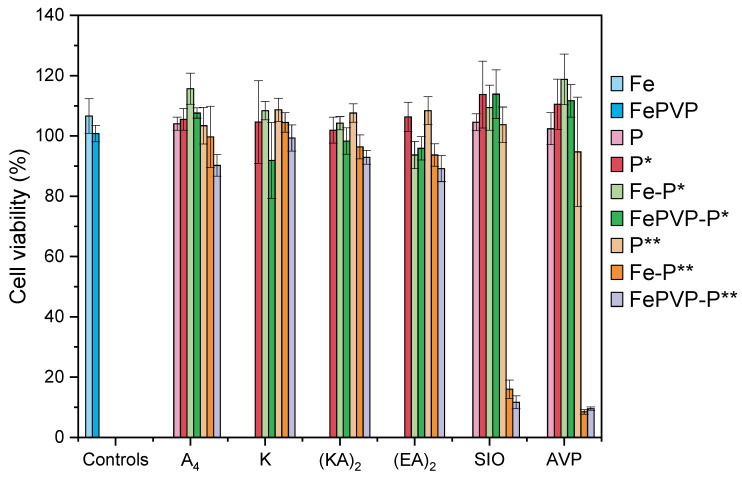
Cell viability of HeLa cells after treatment with peptides (P) or derived IGPNs as determined by the CellTiter-Glo^®^ Luminescent Cell Viability Assay. HeLa cells were incubated for 72 h with Fe, FePVP, SIO, SIO*, SIO **, AVP, AVP*, AVP**, SIO**, Fe-SIO**, FePVP-SIO**, AVP**, Fe-AVP**, or FePVP-AVP** at doses corresponding to 10 μM peptide content (or Fe^3+^ in case of Fe and FePVP).

The general cytotoxicity of IGPNs as well as the ability to induce biological effects by delivering bioactive peptides were evaluated by the CellTiter-Glo assay in HeLa cells (Figure 9). The two pro-apoptotic peptides SIO and AVP were selected as bioactive peptide cargos. FeCl_3_ solution (Fe), the complex of iron and PVP (FePVP), as well as free peptides (unmodified and coupled with gallic acid) were used as controls at the same concentration (10 μM). The viability of cells treated with the model peptides A_4_, A_4_*, and A_4_** indicate that the integration of gallic acid did not affect the tolerability of the derivatives. Moreover, IGPNs assembled from the inactive model peptides did not show obvious toxicity and cell viability levels remained over 80% in all cases. In addition, the pro-apoptotic peptides SIO and AVP, with or without gallic acid modification, were not able to mediate significant cytotoxicity. In contrast, IGPNs assembled from double-GA modified pro-apoptotic peptides (Fe-SIO**, FePVP-SIO**, Fe-AVP**, and FePVP-AVP**) induced strong tumor cell killing and reduced cell viability below 20%. Additional cell viability experiments with Fe-SIO** and FePVP-SIO** determined that the cytotoxic effects are dose-dependent (Figure S13A). To evaluate the cytotoxicity of IGPNs in another tumor cell line, the model peptide (A4) and proapoptotic peptide (AVP) were chosen for testing in murine neuroblastoma Neuro 2A (N2a) cells. In N2a cells, a pronounced cytotoxic effect was also observed with Fe-AVP** and FePVP-AVP** at a dose corresponding to 10 µM peptide, whereas none of the other samples (free A4 or AVP peptides, A4-based IGPNs, Fe-AVP*, or FePVP-AVP*) showed distinct effects on cell viability. These additional findings support the potential utility of IGPNs based on double GA-modified peptides as effective therapeutic agents against different types of tumor cells.

**Figure 10:**
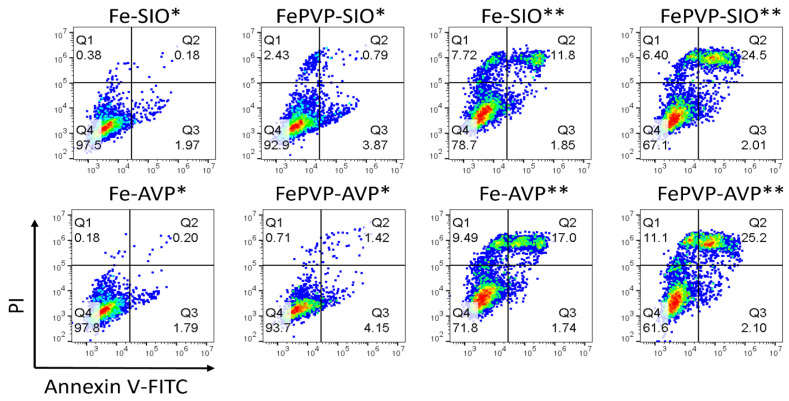
Evaluation of apoptotic events by PI/Annexin V–FITC staining and flow cytometry. HeLa cells were incubated for 24 h with IGPNs at doses corresponding to 10 μM peptide content.

To elucidate the cytotoxicity mechanism of IGPNs more in detail, cell apoptosis assays were carried out via Annexin V and propidium iodide (PI) staining and flow cytometry (Figure 10 and Figure S14). Consistent with the cell viability data, none of the controls or IGPNs assembled from single-GA modified peptides resulted in obvious apoptosis induction; only the cells treated with IGPNs assembled from double-GA modified pro-apoptotic peptides showed an enrichment of Annexin V–FITC positive subpopulations, indicating the occurrence of apoptotic events. These results demonstrate that double-GA modification of bioactive peptides and assembly into IGPNs is a feasible strategy for cytosolic delivery and induction of intracellular biological effects.

## 4. Conclusions

A novel generic strategy for the assembly of peptides into metallo-peptidic nanoparticles is presented. By conjugation of gallic acid (GA) to the side chains of C- and N-terminal lysines, GA modified peptides are generated, which assemble quantitatively with Fe^3+^ or PVP-templated Fe^3+^ (FePVP) into iron–gallic acid peptide nanoparticles (IGPNs). This approach exhibits versatility in its applicability to a wide range of peptides with varying lengths, charges, and sequences. GA functions can readily be integrated into peptide sequences by the presented approach via coupling to C- and N-terminal lysine side chains. IGPNs, especially the double GA-modified FePVP NPs, are stable in water, PBS, and serum-containing medium, but decompose in artificial lysosomal fluid (ALF), which indicates the endo-lysosomal biodegradability of the particles. Although the particle appearance of dry, disperse Fe and FePVP IPGNs appears similar in TEM imaging, the PVP assembly approach is considered to be favorable for higher colloidal stability in an aqueous environment. It could be shown that IGPNs, aided by GA, are able to promote generation of reactive oxygen species (ROS) from H_2_O_2_ via the Fenton reaction. Furthermore, the assembly into IGPNs facilitates cellular uptake of peptides with otherwise poor cellular permeability. Finally, the FePVP IGPNs built from double GA-tagged pro-apoptotic peptides demonstrated potent anti-tumoral activity with apoptosis induction and killing of HeLa and N2a cells.

In summary, the presented data describes a convenient and versatile platform for the cellular delivery of cell-impermeable peptides and highlights the potential utilization for cancer therapy by combining apoptotic peptide cargos with the intrinsic ROS generation of IGPNs.

## Figures and Tables

**Table 1:** Gallic acid (GA)-modified peptides used for the assembly of IGPNs.

Code	Sequence ^a^	Molecular Weight (Da)	Function
K * K **	K(GA) GA-K(GA)	298.3 450.0	minimal peptide motif
A_4_ A_4_ * A_4_ **	AAAA AAAAK(GA) K(GA)AAAAK(GA)	300.2 582.6 862.4	neutral model peptides
(KA)_2_ (KA)_2_ * (KA)_2_ **	KAKA KAKAK(GA) K(GA)KAKAK(GA)	416.3 696.8 977.1	cationic model peptides
(EA)_2_ (EA)_2_ * (EA)_2_ **	EAEA EAEAK(GA) K(GA)EAEAK(GA)	418.4 698.7 979.0	anionic model peptides
SIO SIO * SIO **	QPK QPK(GA) K(GA)QPK(GA)	371.4 523.5 803.8	pro-apoptotic SIO peptides
AVP AVP * AVP **	AVPIAQK AVPIAQK(GA) K(GA)AVPIAQK(GA)	725.9 878.0 1158.3	pro-apoptotic AVP peptides

^a^ Peptide sequences are ordered from N- to C-terminus. * indicates one gallic acid (GA) conjugated to the side chain of the C-terminal lysine (K); ** indicates two GA conjugated to the side chains of the C- and N-terminal K.

## Data Availability

The data presented in this study are available on request from the corresponding author.

## Supplementary Materials

The following supporting information can be downloaded at: https://www.mdpi.com/article/10.3390/pharmaceutics15071789/s1, 1. Methods and Characterization, 2. Supporting Figures (Figure S1. Photographs of IGPN suspensions in water, Table S1. Size (Z-Ave), PdI, zeta-potential (ZP) and Conductivity (Cond) as determined by DLS, Figure S2. Particle size distributions of IGPN suspensions in water, Figure S3. (S)TEM images of Fe-A_4_** and FePVP-A_4_**, Figure S4. XRD measurements of Fe- A_4_*, FePVP- A_4_*, Fe- A_4_**, and FePVP- A_4_**, Figure S5. Determination of free peptides in filtrated solutions via RP–HPLC, Figures S6 and S7. UV–Vis spectrometry of IGPNs and decomposed IGPNs, Figure S8. Thermogravimetric analysis of Fe-A_4_** and FePVP-A_4_**, Figure S9. Cellular uptake of carboxyfluorescein-labeled peptides determined by flow cytometry, Figures S10–S11. Cellular uptake of carboxyfluorescein-labeled peptides determined by confocal laser scanning microscopy (CLSM), Figure S12. Determination of intracellular ROS formation, Figure S13. Cell viability determined by CellTiter-Glo Assay, Figure S14. Evaluation of apoptotic events by PI/Annexin V–FITC staining and flow cytometry), 3. Analytical data (3.1 RP–HPLC, 3.2. ^1^H NMR spectra, 3.3. Mass spectrometry).
