# Supplementary material for: Iron-Gallic Acid Peptide Nanoparticles as a Versatile Platform for Cellular Delivery with Synergistic ROS Enhancement Effect
Source: Pharmaceutics. 2023 Jun 21;15(7):1789. doi: 10.3390/pharmaceutics15071789 (PMC10385416; doi:10.3390/pharmaceutics15071789)
Supplement: Supplementary file 1 [file pharmaceutics-15-01789-s001.zip › pharmaceutics-2402148-supplementary.pdf]

pharmaceutics

Supporting Information

Iron-Gallic Acid Peptide Nanoparticles as a Versatile Platform for Cellular Delivery with Synergistic ROS Enhancement Effect

Faqian Shen,[1] Yi Lin,[1] Miriam Höhn,[1] Xianjin Luo,[1] Markus Döblinger,[2] Ernst Wagner,[1] Ulrich Lächelt[1,3,*]

[1] Pharmaceutical Biotechnology, Department of Pharmacy, Center for NanoScience (CeNS), LMU Munich, 81377 Munich, Germany

[2] Department of Chemistry, LMU Munich, 81377 Munich, Germany

[3] Department of Pharmaceutical Sciences, University of Vienna, 1090 Vienna, Austria

1. Methods and Characterization

Nuclear magnetic resonance (NMR): ^{1}H-NMR spectroscopy was performed with an Advance III HD 400 MHz Bruker BioSpin (400 MHz) with CryoProbe™ Prodigy probe head. Each sample was prepared by dissolving 5-7 milligram of the material in 600 µL *D_2O* or *DMSO-d_6* in NMR tubes (Hilgenberg, standard 5 mm).

MALDI-TOF mass spectrometry: MALDI-TOF mass spectra were measured with a Autoflex II mass spectrometer (Bruker Daltonics, Germany). The matrix solution was composed of 10 mg/mL Super-DHB (90/10 m/m mixture of 2,5-dihydroxybenzoic acid and 2-hydroxy-5-methoxybenzoic acid) in 69.93/30/0.07 (v/v/v) H_2O/acetonitrile/trifluoroacetic acid. 1.5 µL of matrix solution was spotted on a MTP AnchorChip (Bruker Daltonics, Germany). After crystallization of the matrix solution, 1.5 µL of sample solution (1 mg/mL in water) was added onto the matrix spot. Data was recorded either in positive or negative ion mode, depending on the chemical structure.

ESI mass spectrometry: ESI mass spectra were recorded with a Thermo scientific LTQ FT Ultra Fourier transform ion cyclotron with IonMax source. All samples were dissolved in water or 30 % acetonitrile in water at a concentration of 1 mg/mL.

Analytical reversed-phase high performance liquid chromatography (RP- HPLC): RP-HPLC was carried out with a VWR-Hitachi Chromaster 5160 pump system VWR-Hitachi Chromaster 5260 autosampler and a VWR-Hitachi Chromaster 5430 diode array detector (VWR, Darmstadt, Germany) at 280 nm detection wavelength. YMC Hydrosphere C18 (YMC Europe, Dinslaken, Germany) and Waters Sunfire C18 (Waters, Milford, MA, USA) were used as columns. A gradient from 1 % to 100 % acetonitrile in water containing 0.1 % trifluoroacetic acid in 30 min was applied.

Transmission electron microscopy (TEM): For transmission electron microscopy (TEM), scanning transmission electron microscopy (STEM) and energy dispersive X-ray (EDX) spectroscopy, a Titan Themis (FEI) equipped with a Super-X EDX detector, operated at 300 kV was used. Samples were prepared by drying sample droplets on a plasma-activated thin carbon film supported by a copper grid.

X-ray diffraction (XRD): Powder X-ray diffraction (PXRD) measurements were carried out on a Bruker D8 Discover with Ni-filtered Cu Kα radiation and a LynxEye position-sensitive detector in Bragg-Brentano geometry. Kβ radiation was attenuated with a 0.0125 mm Ni filter. All samples were prepared by fixating the dried samples between two polymer foils.

X-ray photoelectron spectroscopy (XPS): The XPS measurements were carried out by using a VSW TA10 X-ray source providing non- monochromatized Al Kα radiation (hν = 1486.6 eV) set at 15 mA and 12 kV and a VSW HA100 hemispherical analyzer. The spectra were recorded with a pass energy of 22 eV and a dwell time of 0.1 s per measurement point. The samples were applied onto a silica wafer and subjected to overnight oven drying, resulting in the formation of a residual dry film on the wafer.

Dynamic light scattering (DLS) and zeta-potential measurements: DLS and zeta potential were measured using the Nano-ZS Zetasizer equipped with DTS-1070 folded capillary cuvettes (Malvern Instruments, Malvern, Worcestershire, United Kingdom). All samples were dispersed in deionized water and measured three times with at least six subruns to determine the z-average and polydispersity index (PdI). Zeta potential measurements were performed in 10 mM NaCl in triplicates with 10–15 subruns.

Thermogravimetric analysis (TGA): Thermogravimetric analysis (TGA): TGA was carried out with a thermo-microbalance (Netzsch, STA 449 C Jupiter) by applying a heating rate of 10 °C/min from room temperature up to 900 °C. Approximately 10 mg of material was heated under synthetic air (N_2/O_2 mixture).

UV-Vis spectroscopy: UV-Vis measurements were carried out using a Cary 3500 UV-Vis multicell spectrophotometer system. All samples were diluted with the respective solvent to a total volume of 1 mL or 3 mL.

2. Supporting Figures

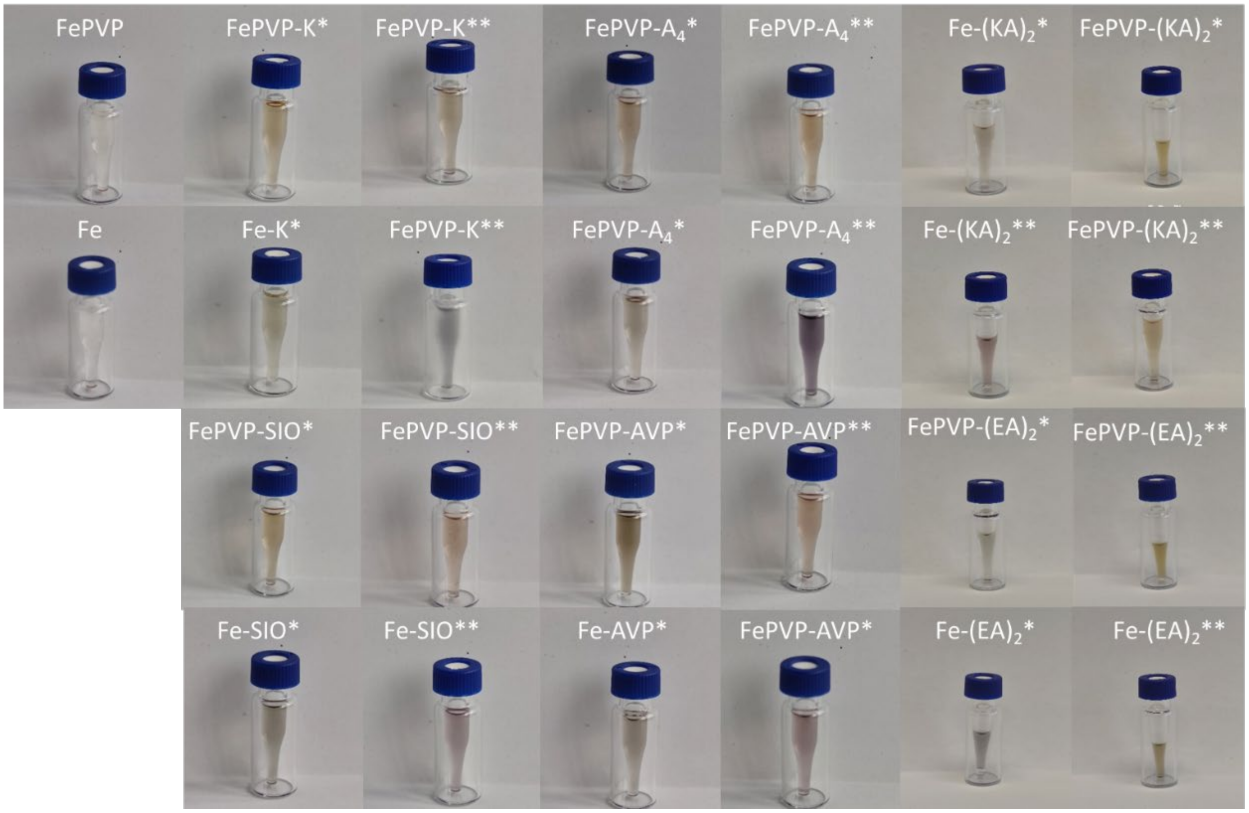

Figure S1. Photographs of IGPN suspensions in water.

Table S1. Size (Z-Ave), PdI, zeta-potential (ZP) and Conductivity (Cond) as determined by DLS

Nanoparticles	*Z-Ave (d.nm)*	*PdI*	*ZP (mV)*	*Cond (mS/cm）*
Fe-K*	115.3±0.26	0.46±0.01	-25±2.48	1.19±0.05
FePVP-K*	154.9±2.25	0.29±0.04	-0.3±0.2	1.4±0.08
Fe-K**	264.7±36.4	0.61±0.07	-16.8±3.35	1.21±0.05
FePVP-K**	174.7±2.22	0.45±0	0.88±0.12	1.55±0.08
$Fe\text{-}A_4*$	310.7±39.29	0.37±0.07	-3.92±0.21	1.4±0.06
$FePVP\text{-}A_4*$	188.6±1.7	0.26±0.01	2.54±0.04	1.36±0.07
$Fe\text{-}A_4**$	111.51±2.68	0.15±0.03	-11.9±2.77	1.14±0.05
$FePVP\text{-}A_4**$	207.3±0.24	0.27±0.01	-5.06±0.85	1.07±0.05
$Fe\text{-}(KA)_2*$	351.7±25.45	0.23±0.09	-2.77±0.2	1.19±0.05
$FePVP\text{-}(KA)_2*$	258.3±2.14	0.06±0.03	2.17±0.1	1.71±0.08
$Fe\text{-}(KA)_2**$	171.2±32.91	0.27±0.04	-3.6±3.42	1.25±0.07
$FePVP\text{-}(KA)_2**$	239.9±0.49	0.06±0.05	2.83±0.25	1.53±0.07
$Fe\text{-}(EA)_2*$	384.4±22.35	0.38±0.05	-2.13±0.76	1.16±0.05
$FePVP\text{-}(EA)_2*$	219.5±1.22	0.11±0.04	-0.77±0.11	1.79±0.09
$Fe\text{-}(EA)_2**$	273.3±27.16	0.35±0.02	-18.6±2.27	1.1±0.05
$FePVP\text{-}(EA)_2**$	255.2±1.13	0.06±0.04	1.16±0.2	1.8±0.09
Fe-SIO*	107.3±0.61	0.38±0.02	-11.2±0.17	1.19±0.06
FePVP-SIO*	142.2±4.82	0.34±0.04	2.74±0.53	1.48±0.08
Fe-SIO**	183.4±7.29	0.34±0.02	-10.7±1.48	1.12±0.05
FePVP-SIO**	181±93.98	0.35±0.14	-13.1±1.37	1.18±0.05
Fe-AVP*	83.45±3.01	0.49±0.07	-21.6±0.72	1.24±0.05
FePVP-AVP*	118.97±1.85	0.37±0.01	-25.2±2.06	1.34±0.05
Fe-AVP**	179±1.78	0.27±0.04	-2.53±0.39	1.12±0.05
FePVP-AVP**	181±93.98	0.35±0.14	-13.1±1.37	1.14±0.06

* indicates one gallic acid (GA) conjugated to the side chain of the C-terminal lysine (K); ** indicates two GA conjugated to the side chains of the C- and N-terminal K.

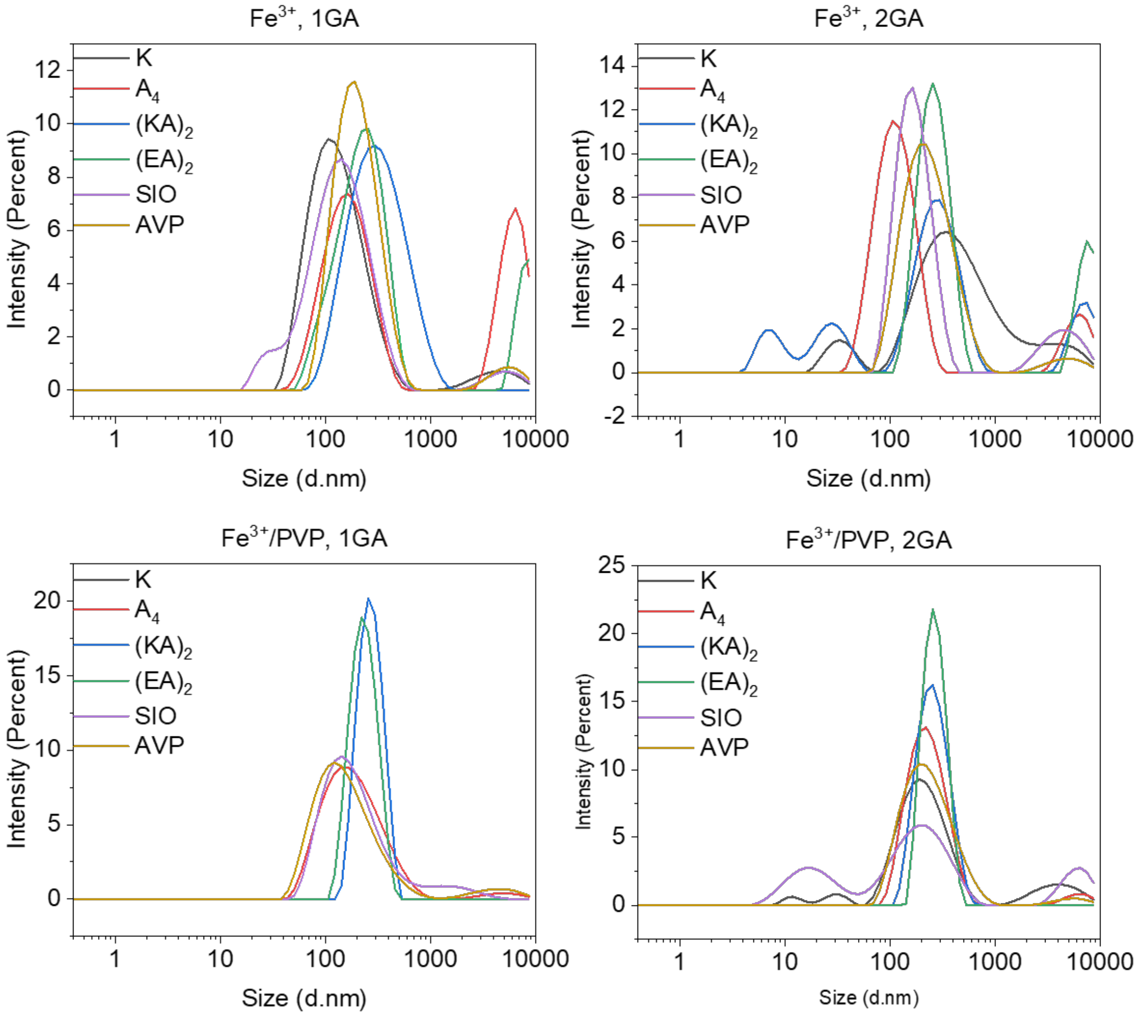

Figure S2. Particle size distributions of IGPN suspensions in water.

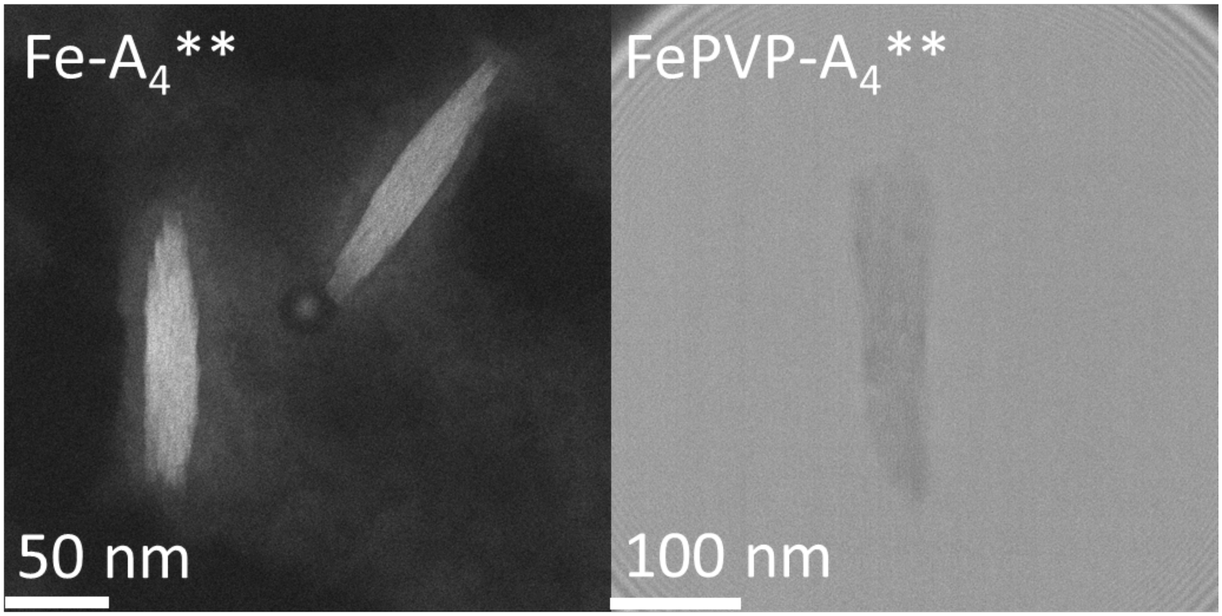

Figure S3. (S)TEM images of **Fe-A$_4$**, FePVP-A$_4$****.

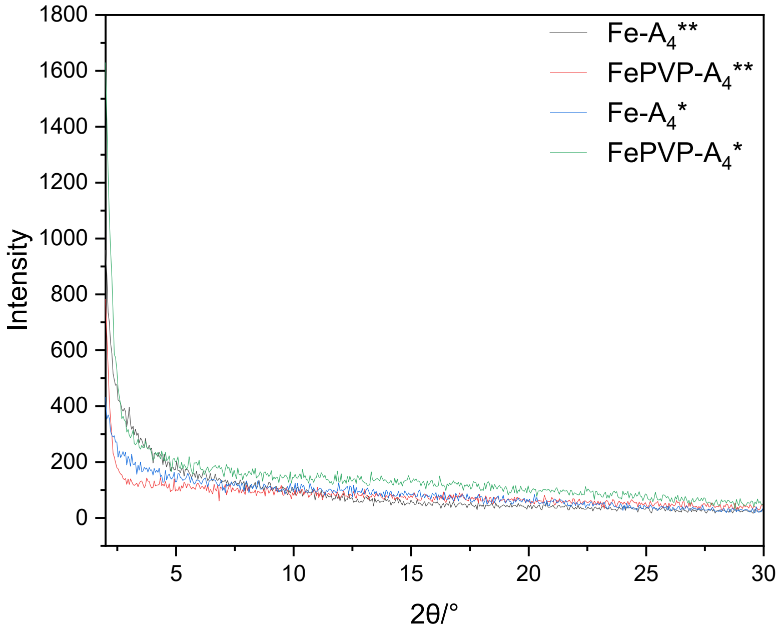

Figure S4. XRD measurements of **Fe- A$_4$*, FePVP- A$_4$*, Fe- A$_4$**, FePVP- A$_4$****.

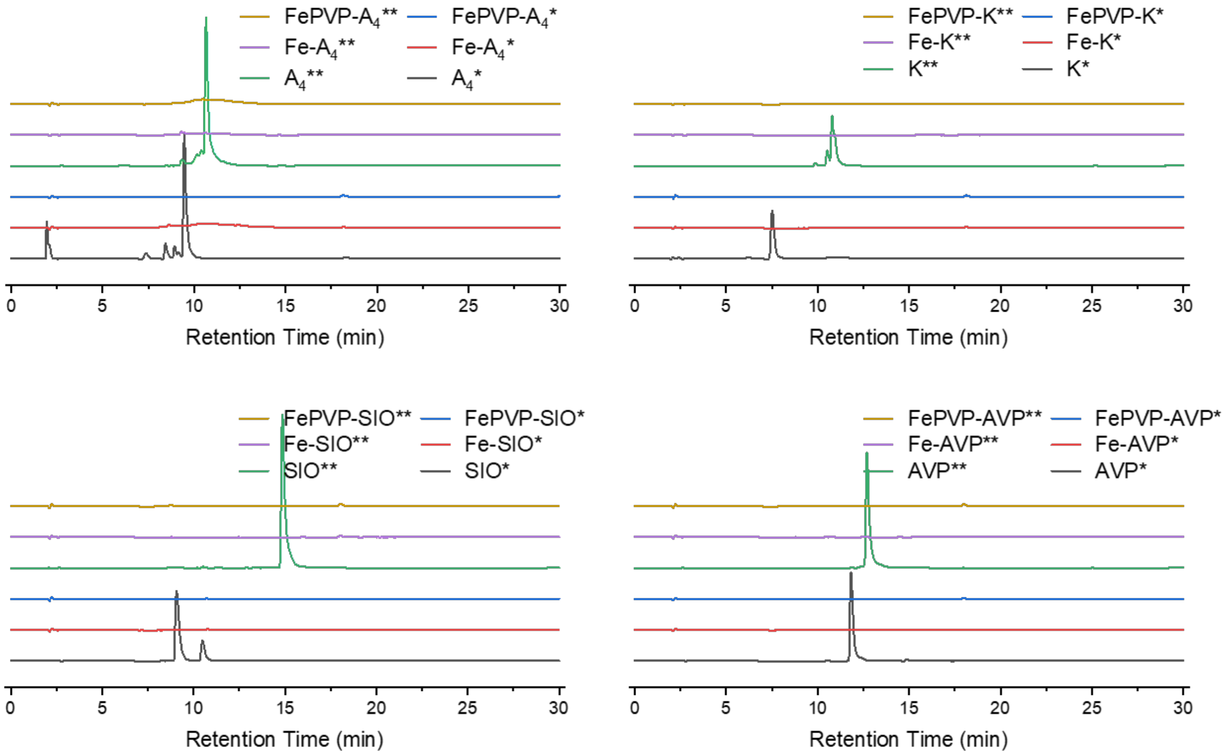

Figure S5. Determination of free peptides in filtrated solutions (Agilent Spin Filter, 10kD) via RP-HPLC. Free peptide solutions (100 µM) served as controls.

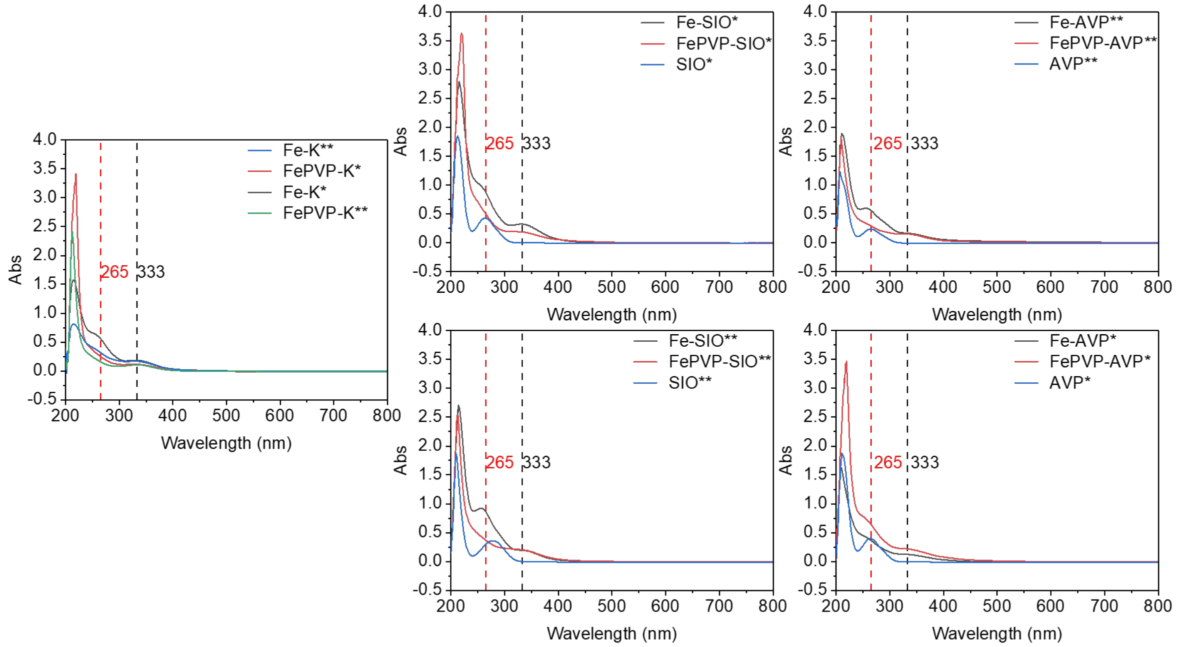

Figure S6. UV-Vis spectrometry of **Fe-K***, **FePVP-K***, **Fe-K****, **FePVP-K****, **SIO***, **Fe-SIO***, **FePVP-SIO***, **SIO****, **Fe-SIO****, **FePVP-SIO****, **AVP***, **Fe- AVP***, **FePVP-AVP***, **AVP****, **Fe-AVP**** and **FePVP- AVP**** after acidic decomposition.

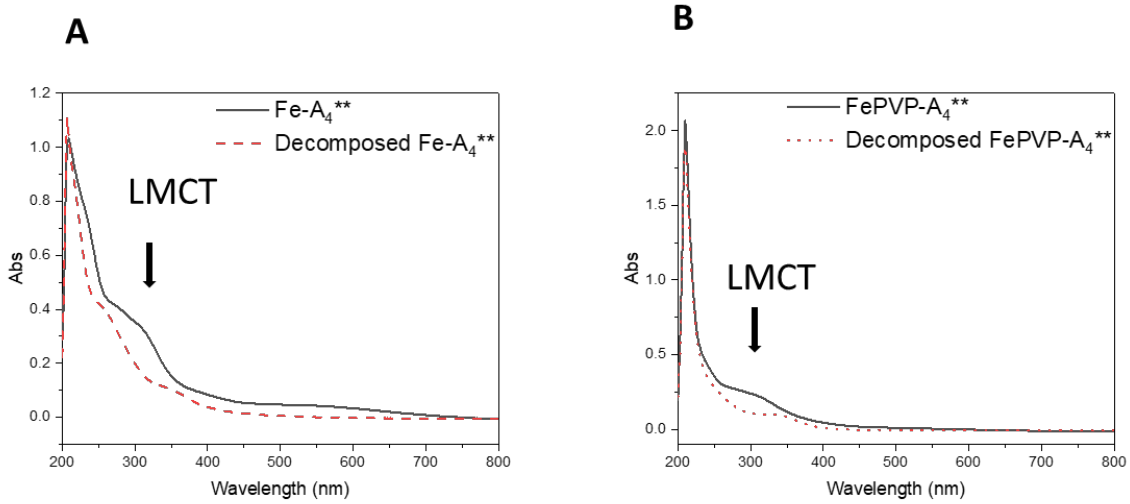

Figure S7. UV-Vis spectrometry. (A) **Fe-A$_4$**** (black) and **Fe-A$_4$**** after acidic decomposition (red). (B) **FePVP-A$_4$**** (black) and **FePVP-A$_4$**** after acidic decomposition (red). "LMCT" indicates the characteristic ligand-to-metal charge transfer band.

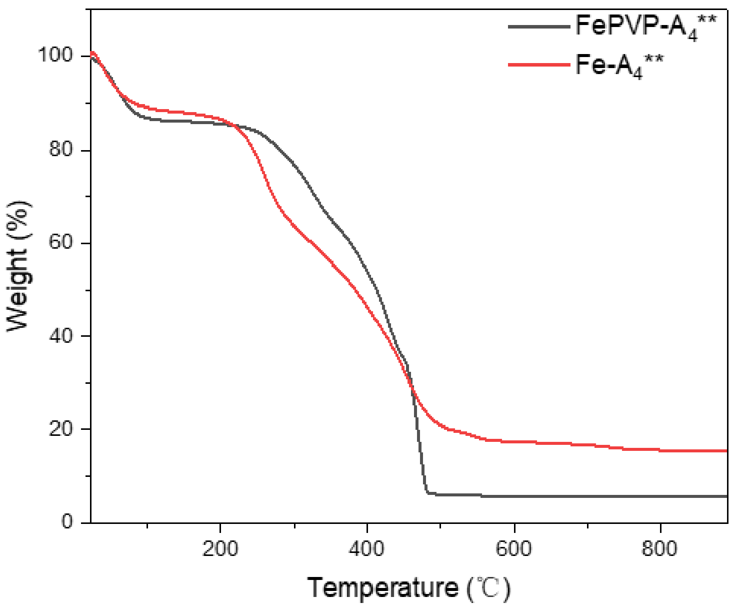

Figure S8. Thermogravimetric analysis of **Fe-A$_4$** and FePVP-A$_4$****.

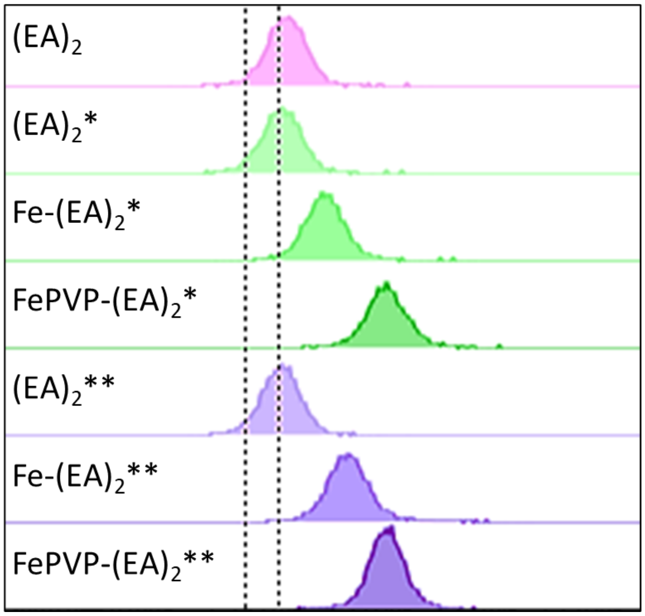

Figure S9. Cellular uptake of carboxyfluorescein-labeled peptides determined by flow cytometry. HeLa cells were treated with peptides or derived **IGPNs** for 4 h.

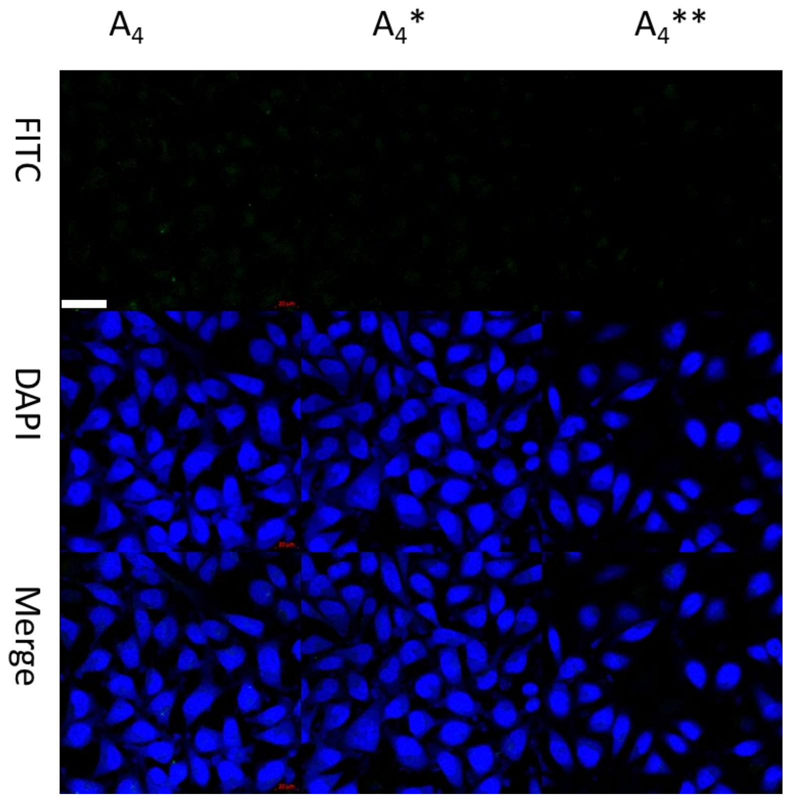

Figure S10. Cellular uptake of carboxyfluorescein-labeled peptides determined by confocal laser scanning microscopy (CLSM). Hela cells were incubated with free **A_4, A_4*, A_4**** for 4 h. Nuclei were stained with DAPI (blue), FITC indicates carboxyfluorescein fluorescence (green). Scale bar: 40 µm.

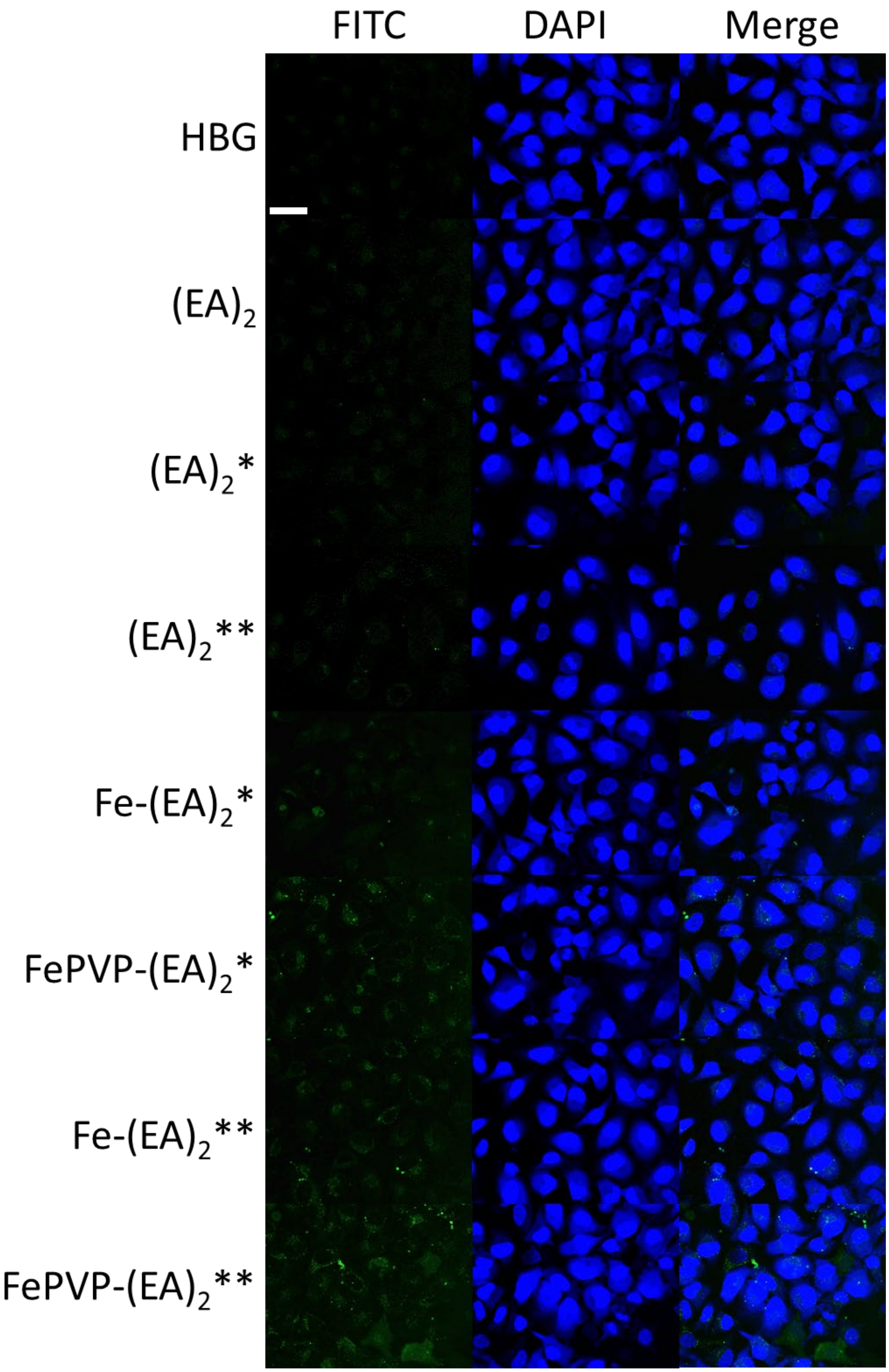

Figure S11. Cellular uptake of of carboxyfluorescein-labeled peptides determined by confocal laser scanning microscopy (CLSM). Hela cells were incubated with **HBG**, **(EA)$_2$**, **(EA)$_2$***,

(EA)$_2$**, **Fe-(EA)$_2$***, **FePVP-(EA)$_2$***, **Fe-(EA)$_2$****, **FePVP-(EA)$_2$**** for 4 hours. Nuclei were stained with DAPI (blue), FITC indicates carboxyfluorescein fluorescence (green). Scale bar: 40 μm.

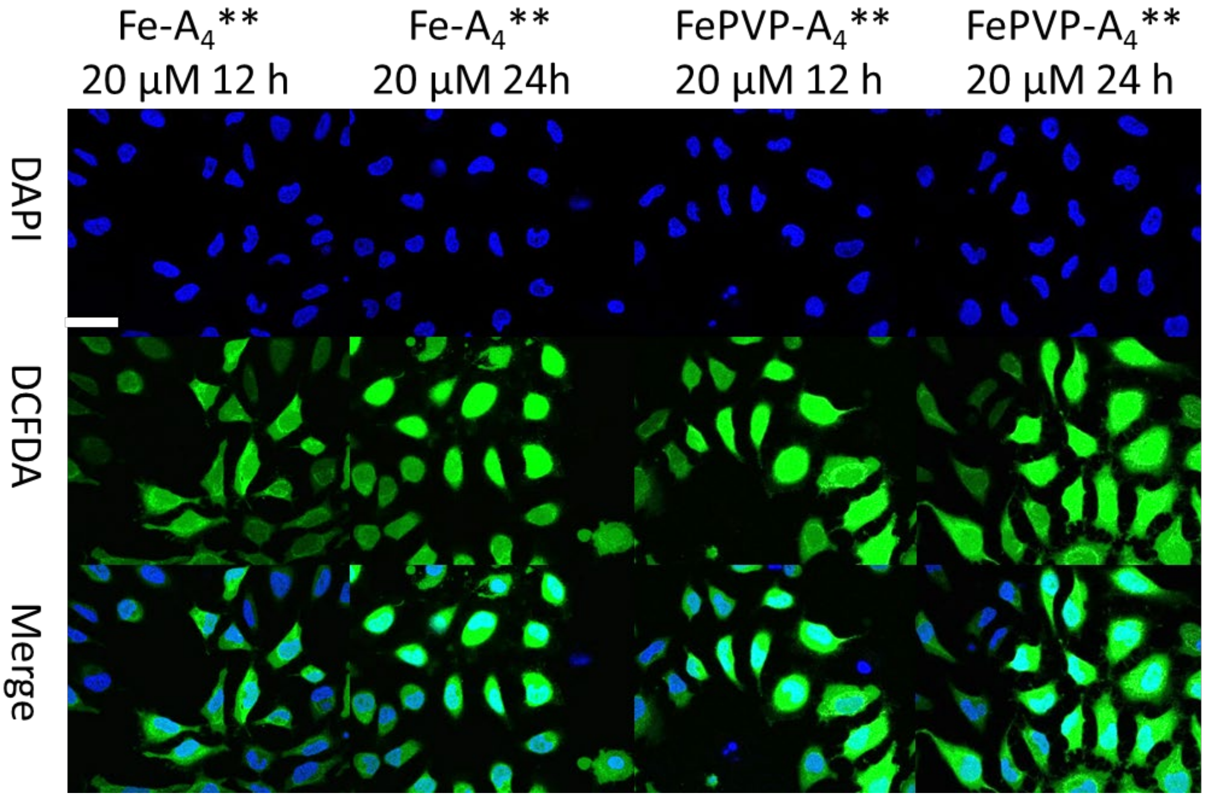

Figure S12. Determination of intracellular ROS formation. Hela cells were incubated with **Fe-A$_4$**** (20 μM) and **FePVP-A$_4$**** (20 μM) for 12 h or 24h, respectively. Nuclei were stained with DAPI (blue) and ROS was detected by DCFDA (green). Scale bar: 40 μm.

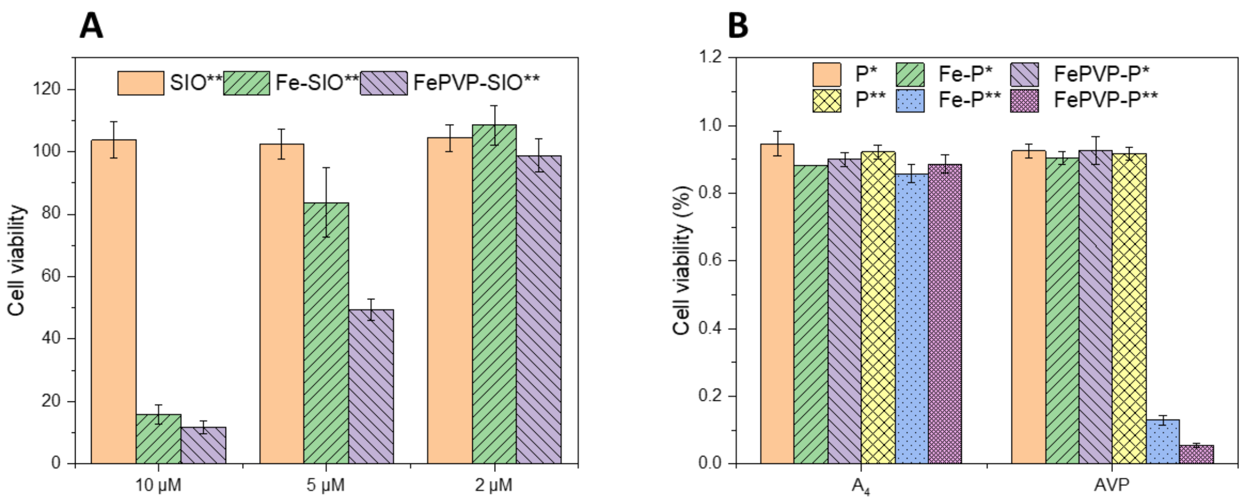

Figure S13. Cell viability determined by CellTiter-Glo Assay. (A) HeLa cells were incubated with **SIO****, **Fe-SIO****and **FePVP-SIO**** (2, 5, and 10 µM peptide content), (B) N2a cells were incubated with **A$_4$***, **A$_4$****, **AVP***, **AVP**** and derived **Fe and FePVP IGPNs** (10 µM peptide content) for 72 h before evaluation via CellTiter-Glo® Luminescent Cell Viability Assay.

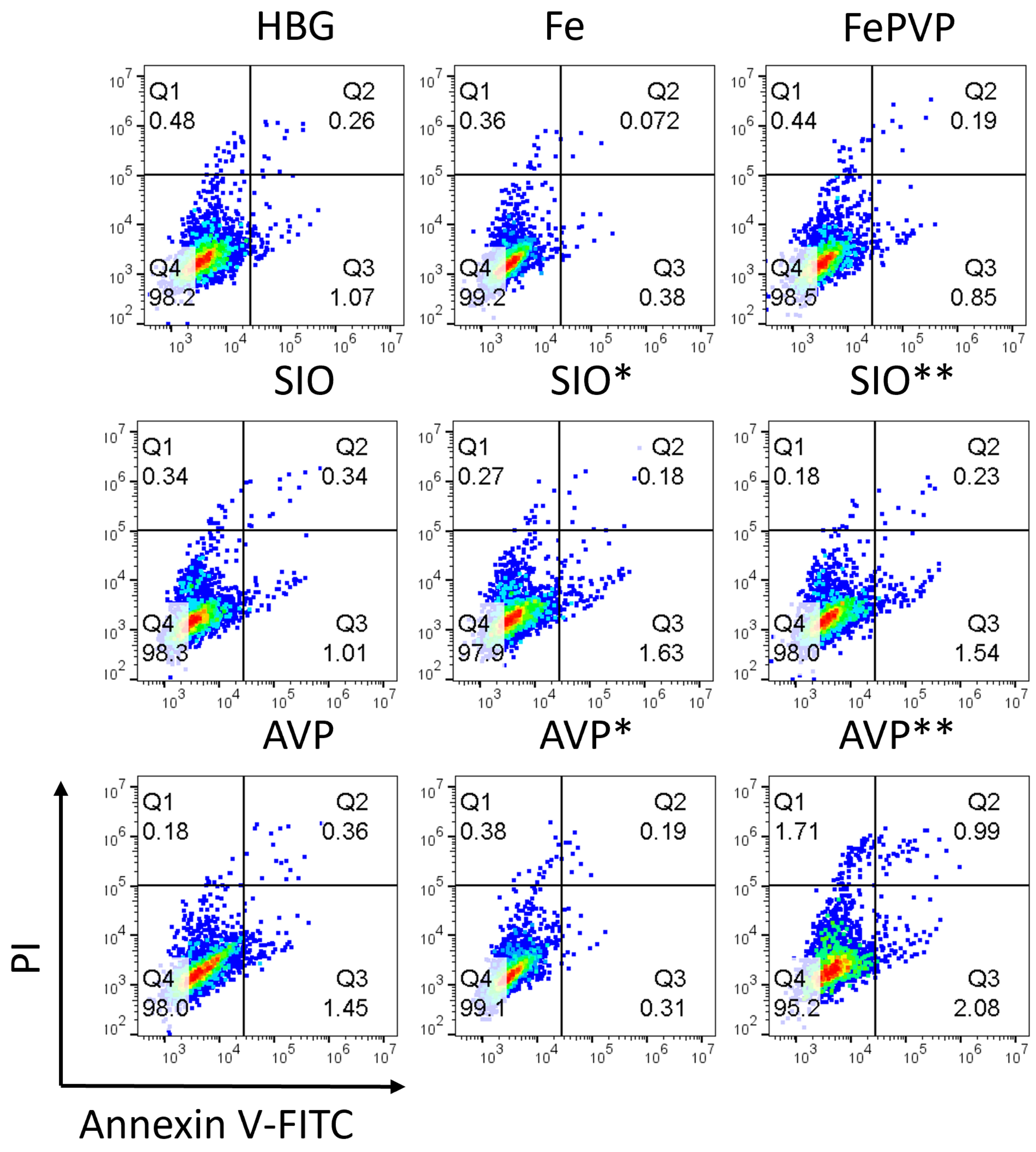

Figure S14. Evaluation of apoptotic events by PI/Annexin V-FITC staining and flow cytometry. HeLa cells were incubated for 24 h with **Fe, FePVP**, **SIO**, **SIO***, **SIO****, **AVP**, **AVP***, **AVP**** at doses corresponding to 10 µM peptide or Fe^{3+} concentrations (in case of Fe and FePVP).

3. Analytical data

3.1 HPLC (RP-C18, detection at 280 nm)

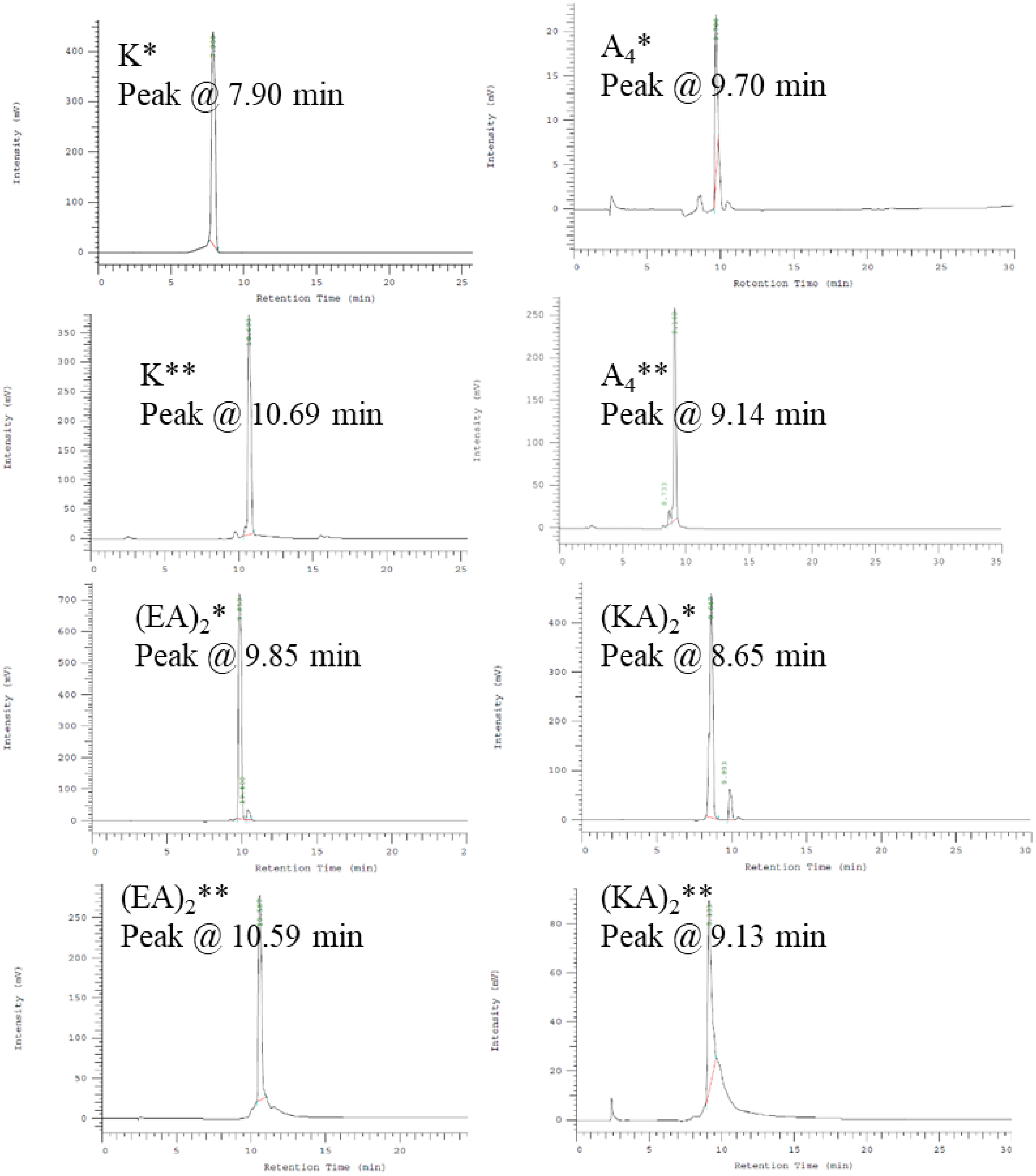

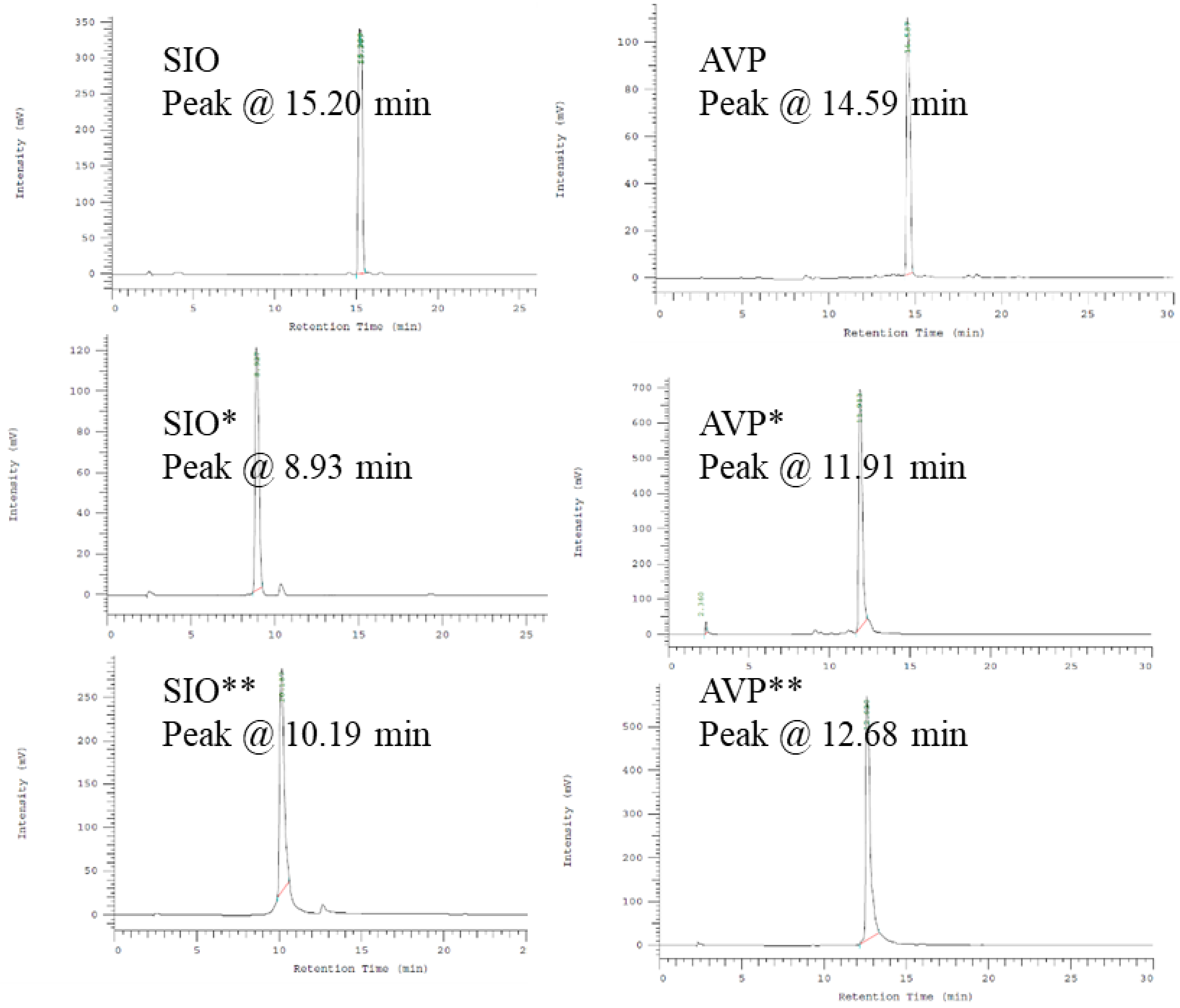
SIO
Peak @ 15.20 min
AVP
Peak @ 14.59 min
SIO*
Peak @ 8.93 min
AVP*
Peak @ 11.91 min
SIO**
Peak @ 10.19 min
AVP**
Peak @ 12.68 min
Intensity (mV)
Retention Time (min)

3.2 ^{1}H NMR spectra

GA(Boc)3-OH

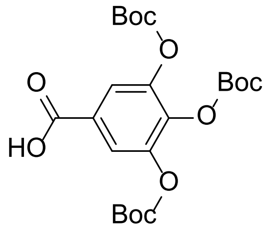

Chemical Formula: $C_{22}H_{30}O_{11}$
Exact Mass: 470.18

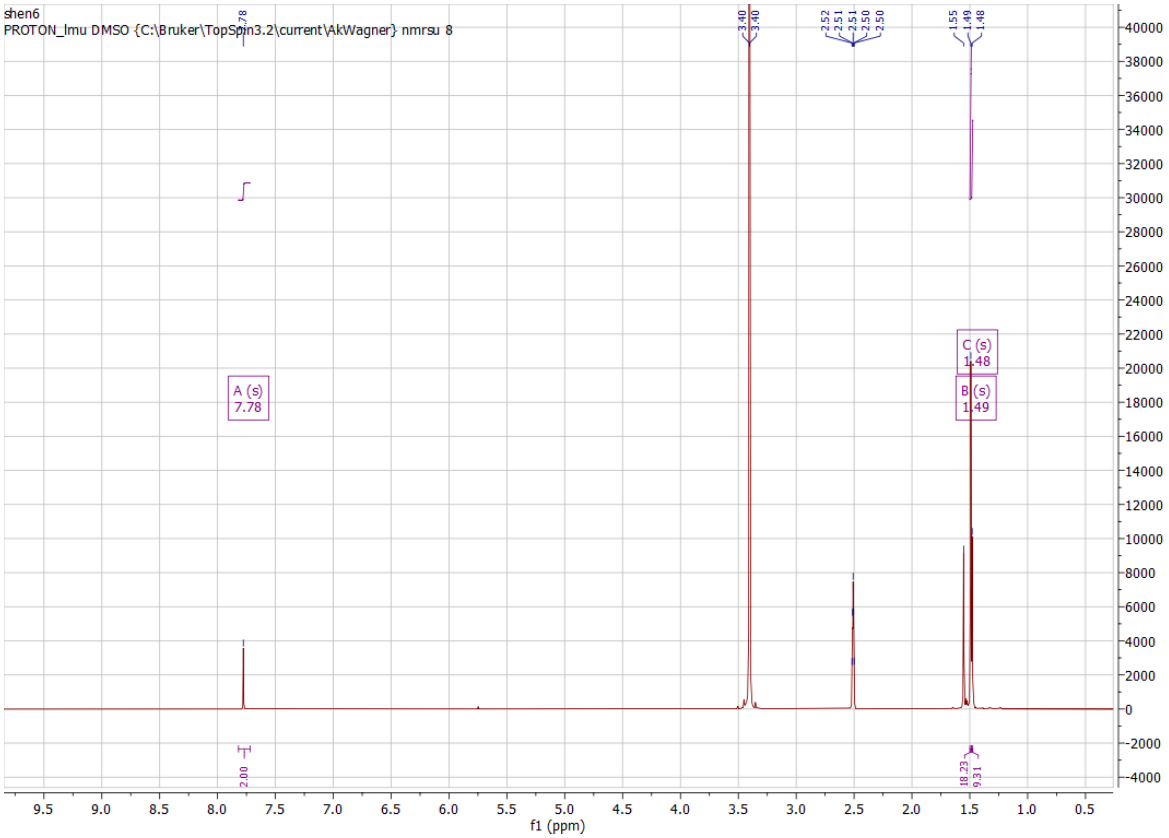

^{1}H NMR (400 MHz, *DMSO-d$_6$*) δ 7.78 (d, J = 0.6 Hz, 2H), 1.49 (s, 18H), 1.48 (s, 9H).

K*

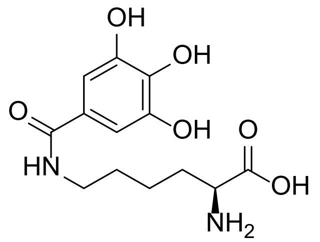

Chemical Formula: $C_{13}H_{18}N_2O_6$
Exact Mass: 298.12

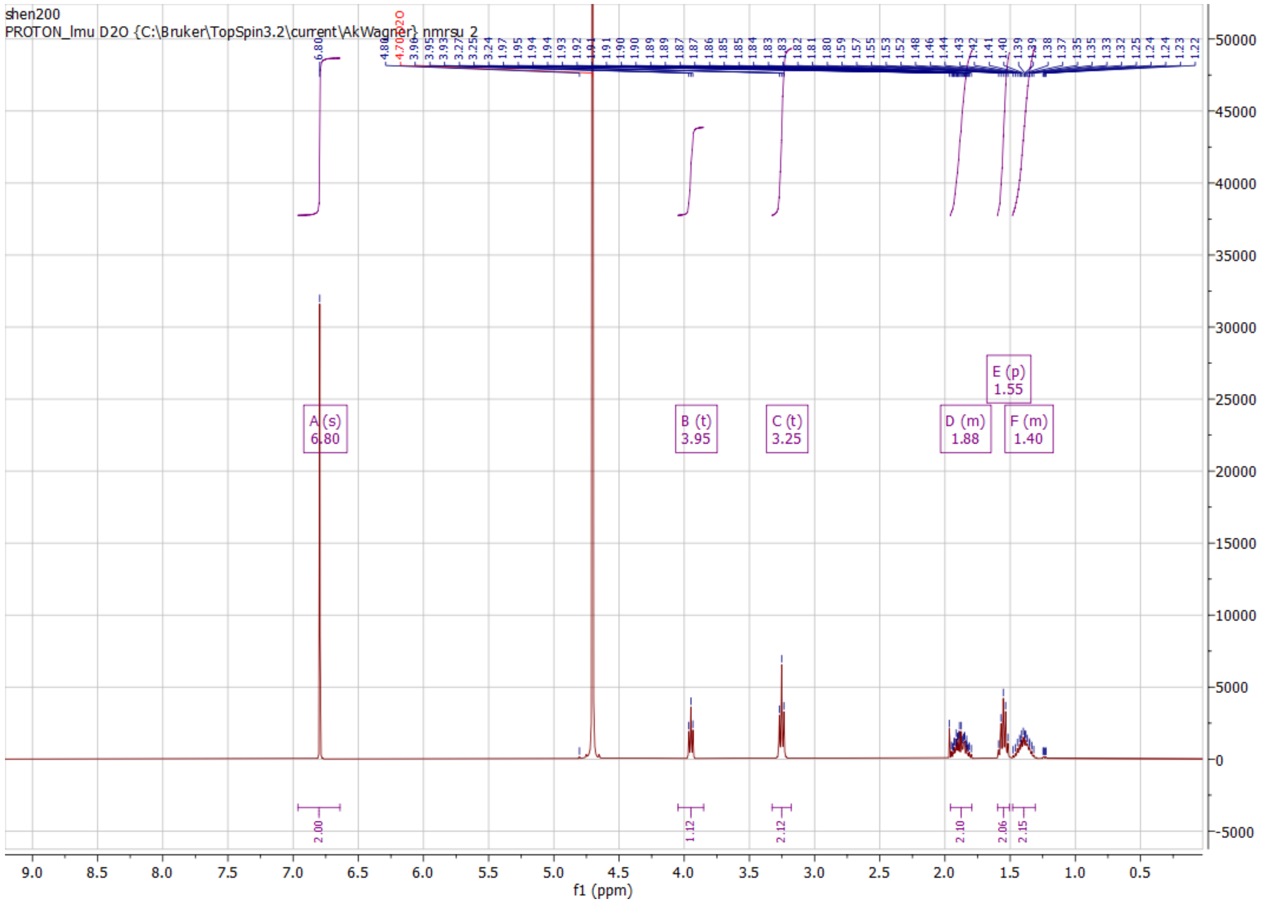

^{1}H NMR (400 MHz, Deuterium Oxide) δ 6.80 (s, 2H), 3.95 (t, *J* = 6.3 Hz, 1H), 3.25 (t, *J* = 6.9 Hz, 2H), 1.96 – 1.79 (m, 2H), 1.55 (p, *J* = 7.2 Hz, 2H), 1.48 – 1.31 (m, 2H).

K**

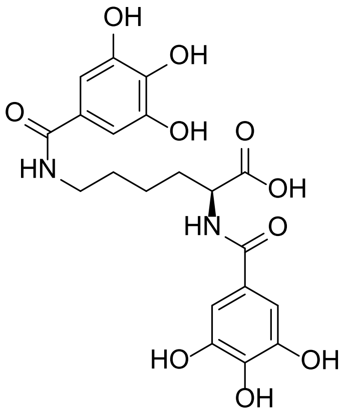

Chemical Formula: $C_{20}H_{22}N_2O_{10}$
Exact Mass: 450.13

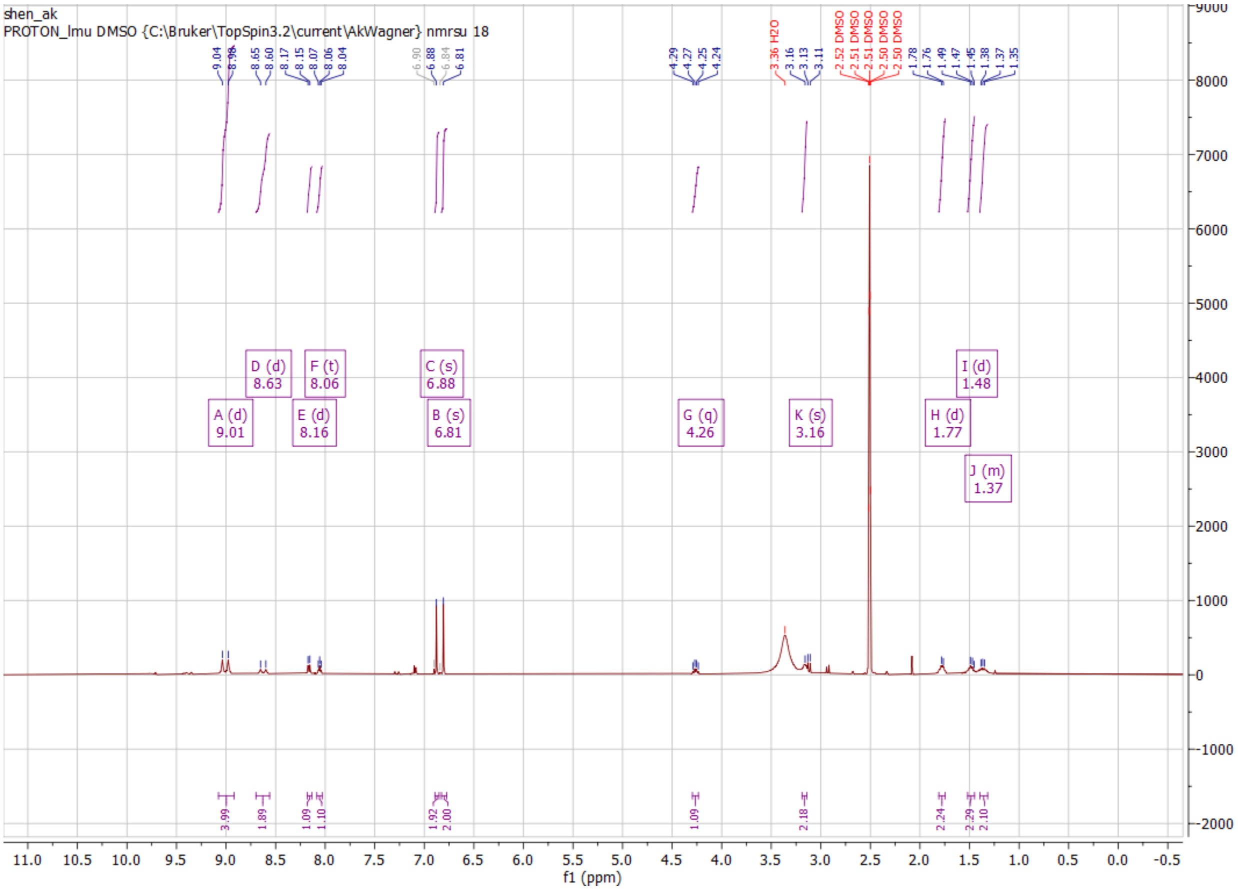

^{1}H NMR (400 MHz, DMSO-d_6) δ 9.01 (d, *J* = 23.4 Hz, 3H), 8.63 (d, *J* = 21.4 Hz, 2H), 8.16 (d, *J* = 7.6 Hz, 1H), 8.06 (t, *J* = 5.5 Hz, 1H), 6.88 (s, 2H), 6.81 (s, 2H), 4.26 (q, *J* = 7.5 Hz, 1H), 3.16 (s, 2H), 1.77 (d, *J* = 7.6 Hz, 2H), 1.48 (d, *J* = 8.3 Hz, 2H), 1.41 – 1.31 (m, 2H).

A4

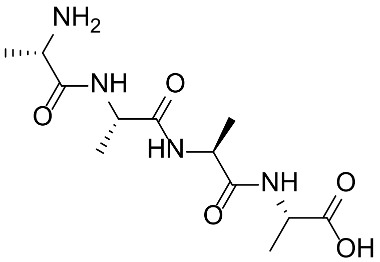

Chemical Formula: $C_{12}H_{22}N_4O_5$
Exact Mass: 302.16

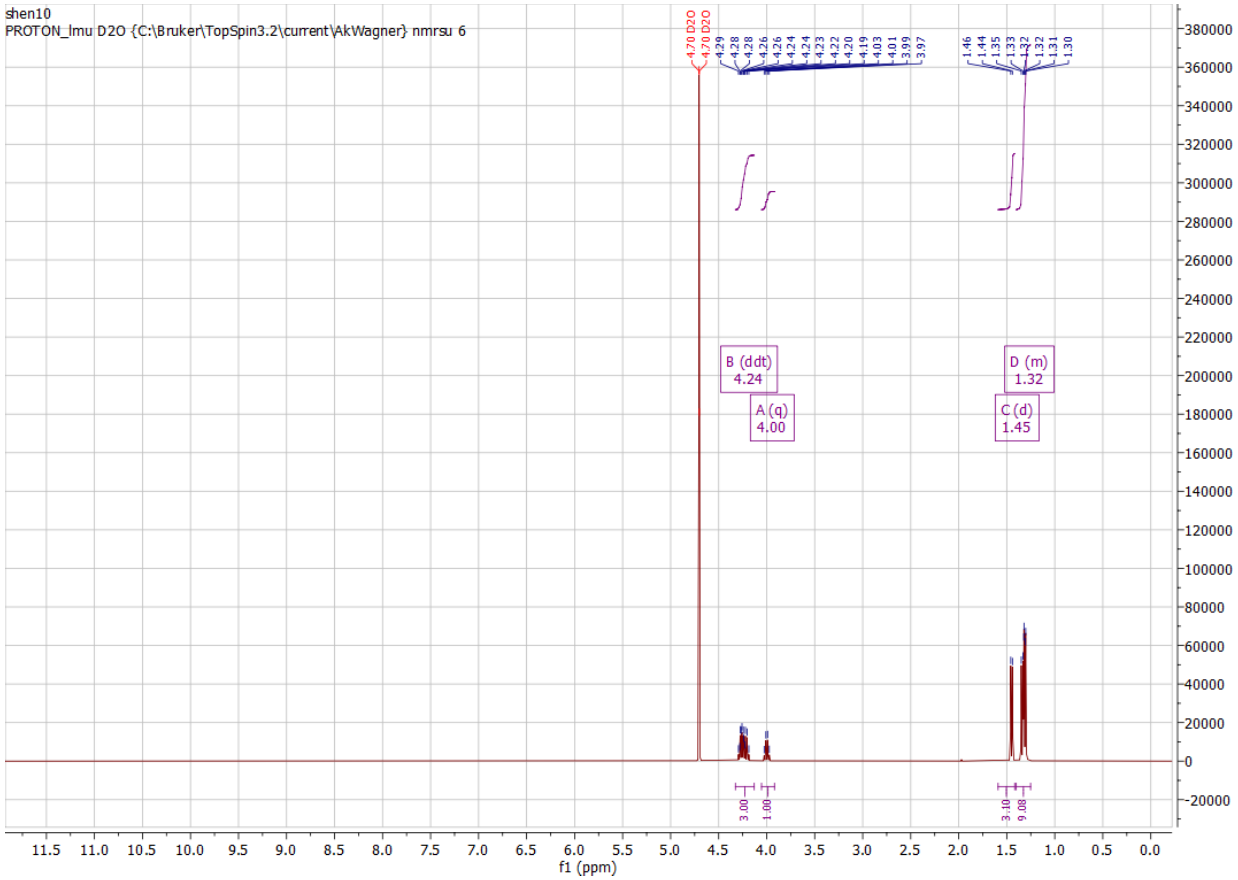

^{1}H NMR (400 MHz, Deuterium Oxide) δ 4.24 (ddt, *J* = 15.9, 14.4, 7.2 Hz, 3H), 4.00 (q, *J* = 7.1 Hz, 1H), 1.45 (d, *J* = 7.1 Hz, 3H), 1.37 – 1.24 (m, 9H).

A_4*

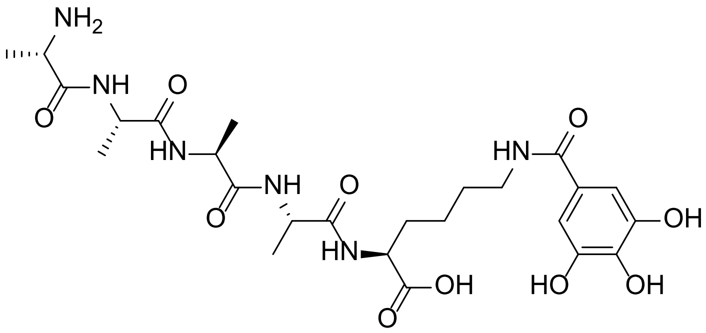

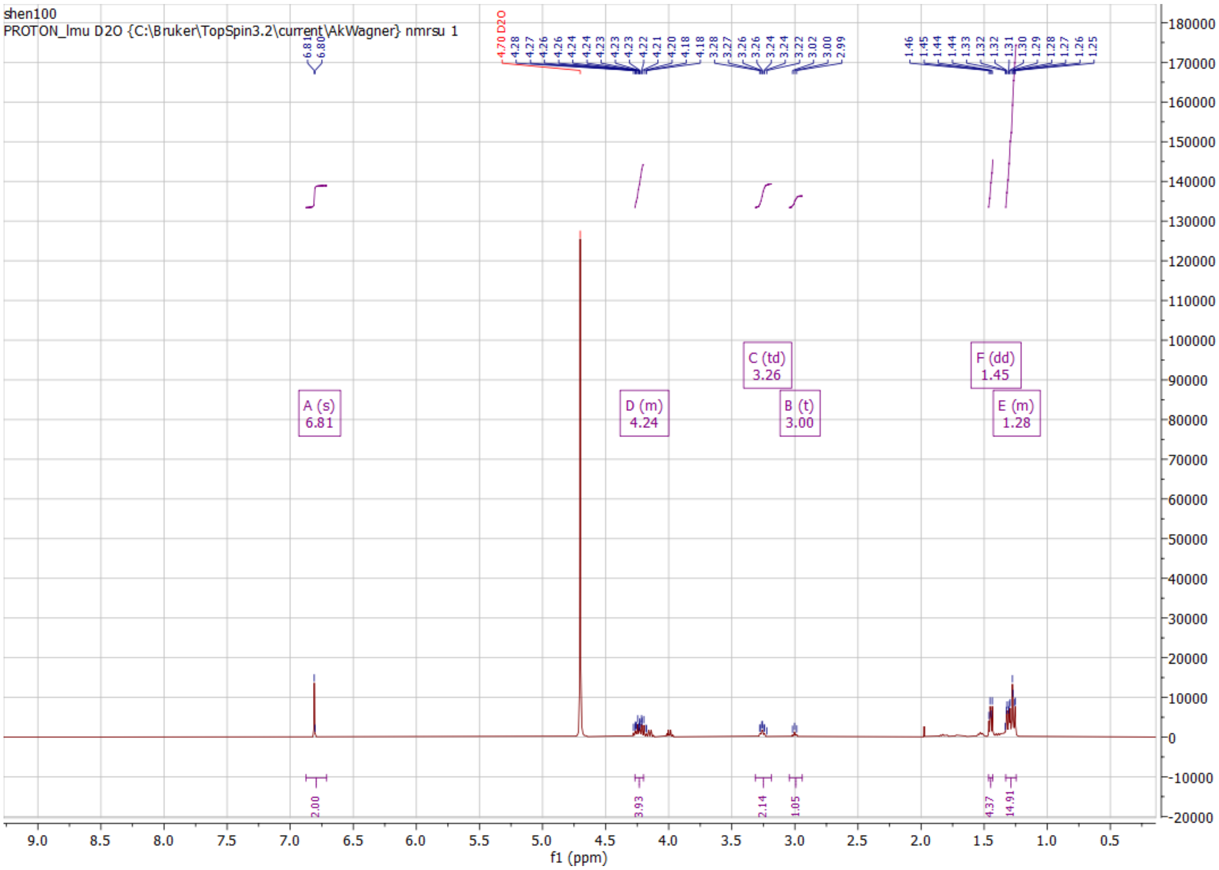

^{1}H NMR (400 MHz, Deuterium Oxide) δ 6.81 (s, 2H), 4.27 – 4.20 (m, 4H), 3.26 (td, *J* = 6.7, 2.3 Hz, 2H), 3.00 (t, *J* = 6.7 Hz, 1H), 1.45 (dd, *J* = 7.1, 3.7 Hz, 4H), 1.33 – 1.25 (m, 15H).

A_4**

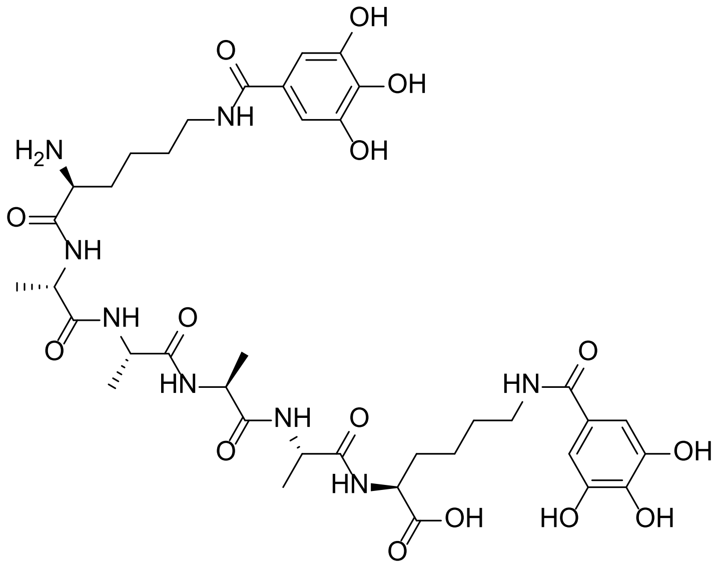

Chemical Formula: $C_{38}H_{54}N_8O_{15}$
Exact Mass: 862.37

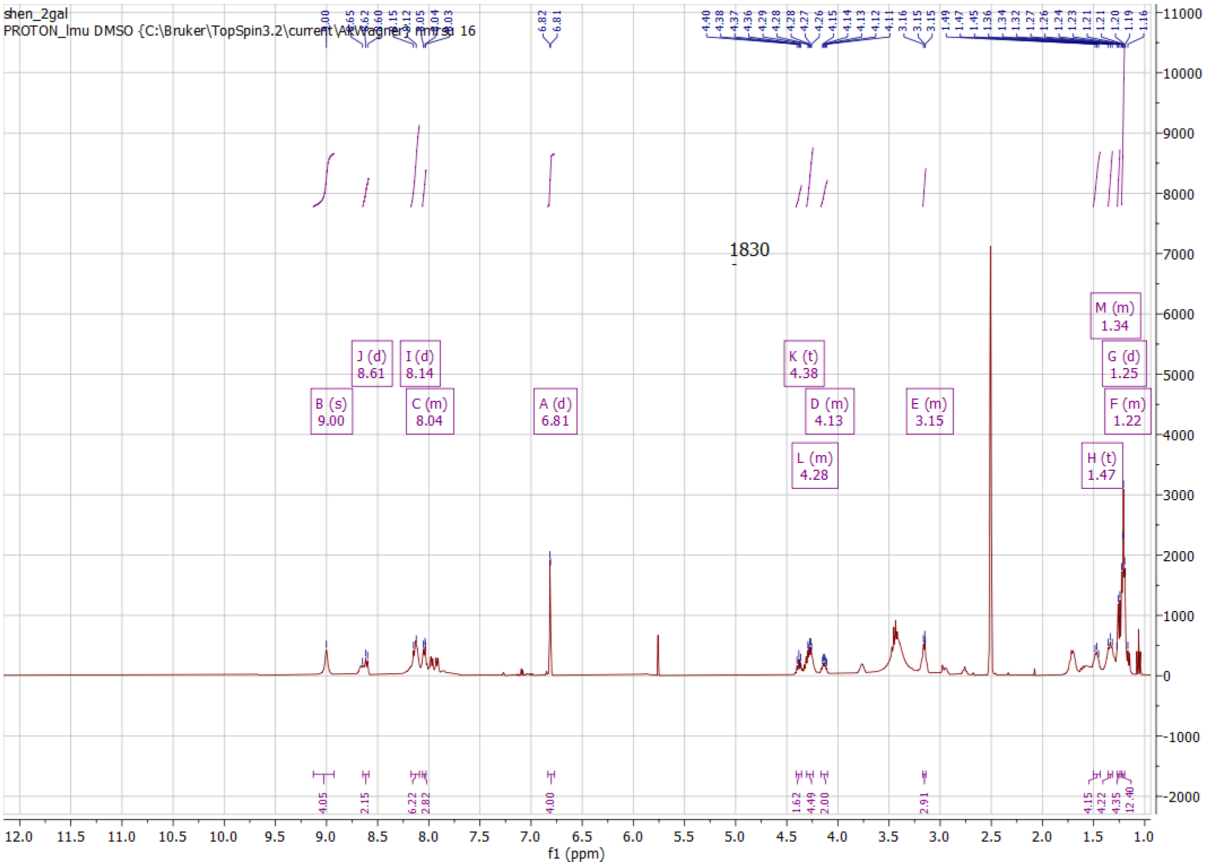

^{1}H NMR (400 MHz, DMSO-d_6) δ 9.00 (s, 4H), 8.61 (d, *J* = 7.3 Hz, 2H), 8.14 (d, *J* = 10.9 Hz, 6H), 8.06 – 8.03 (m, 3H), 6.81 (d, *J* = 2.4 Hz, 4H), 4.38 (t, *J* = 7.2 Hz, 2H), 4.31 – 4.24 (m, 4H), 4.17 – 4.10 (m, 2H), 3.17 – 3.14 (m, 3H), 1.47 (t, *J* = 6.9 Hz, 4H), 1.36 – 1.32 (m, 4H), 1.25 (d, *J* = 7.0 Hz, 4H), 1.23 – 1.20 (m, 12H).

$(KA)_2$*

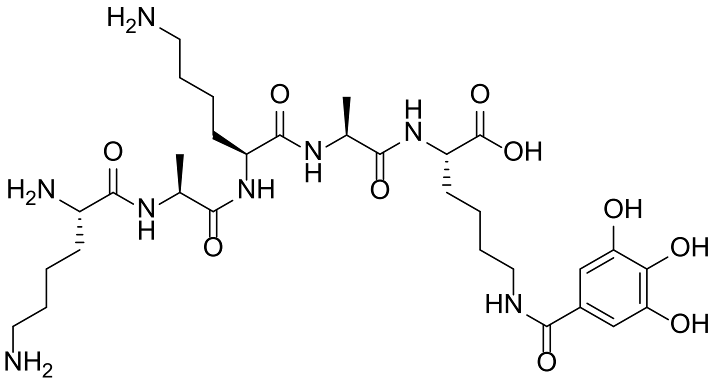

Chemical Formula: $C_{31}H_{52}N_8O_{10}$
Exact Mass: 696.38

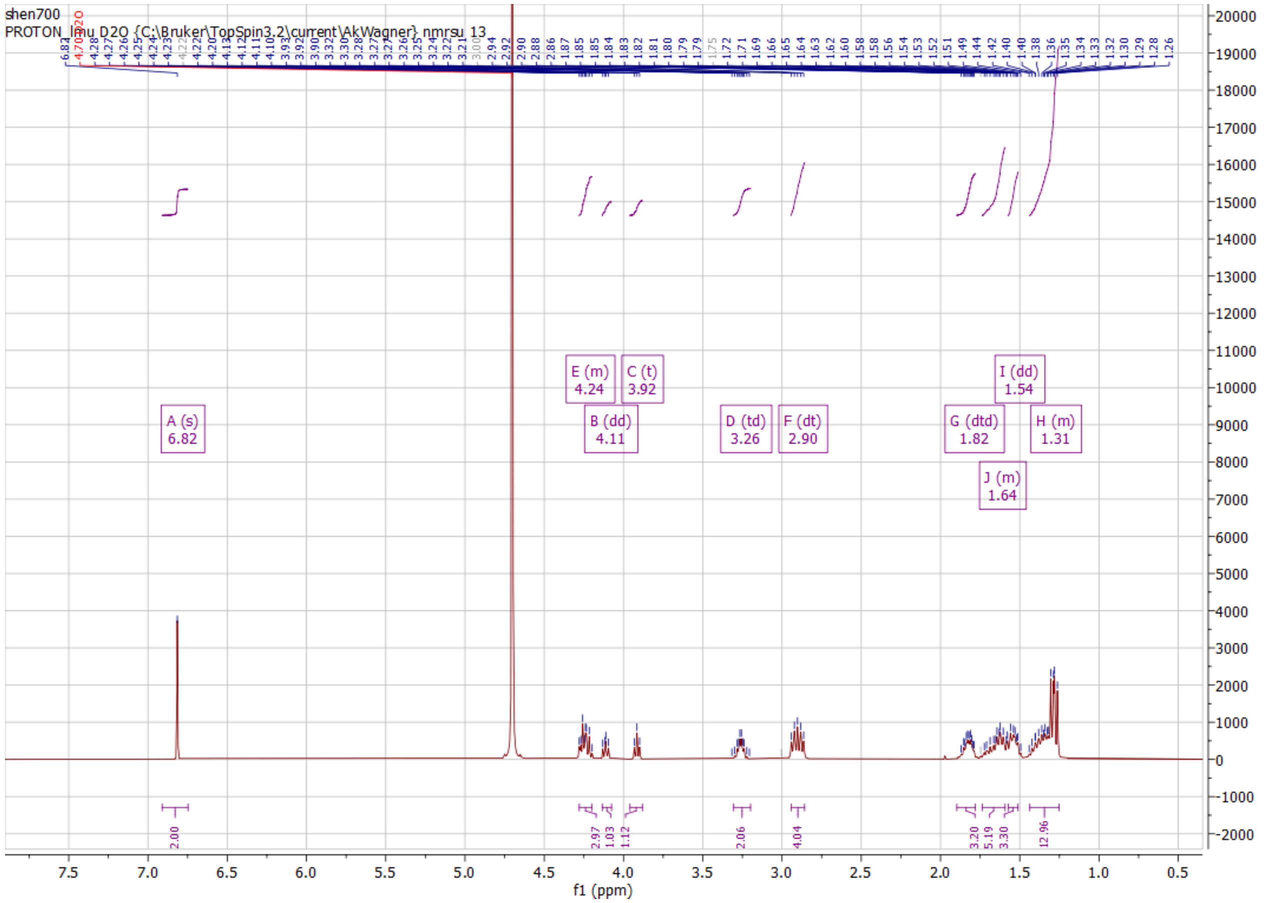

^{1}H NMR (400 MHz, Deuterium Oxide) δ 6.82 (s, 2H), 4.28 – 4.20 (m, 3H), 4.11 (dd, J = 8.0, 6.3 Hz, 1H), 3.92 (t, J = 6.7 Hz, 1H), 3.26 (td, J = 6.7, 3.6 Hz, 2H), 2.90 (dt, J = 15.5, 7.6 Hz, 4H), 1.82 (dtd, J = 9.3, 6.3, 2.8 Hz, 3H), 1.74 – 1.59 (m, 5H), 1.54 (dd, J = 11.2, 7.4 Hz, 3H), 1.44 – 1.25 (m, 13H).

(KA)$_2$**

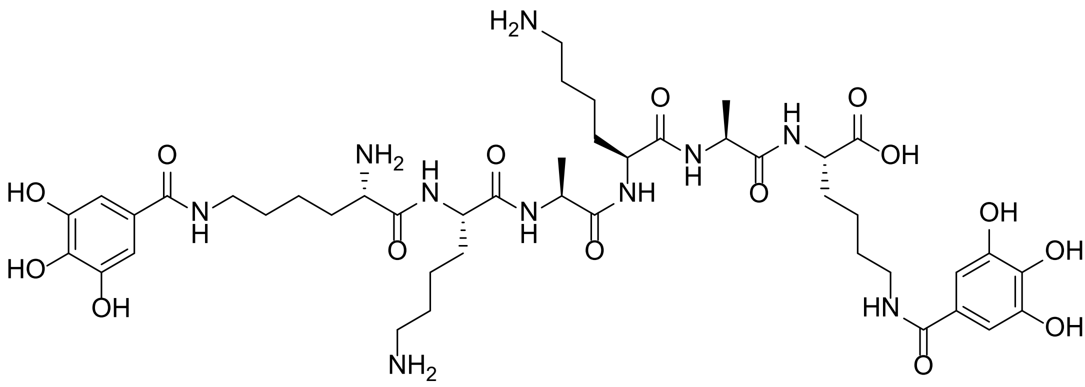

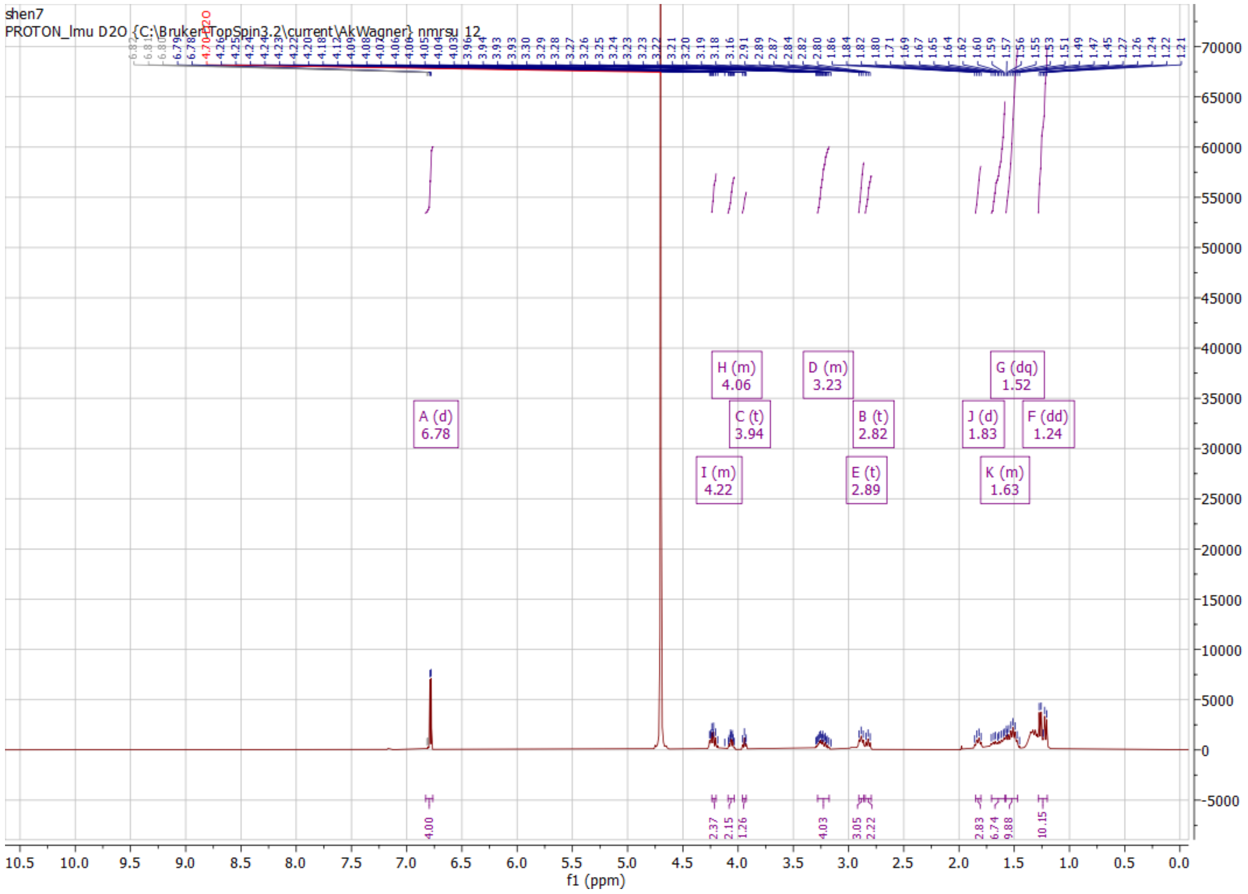

^{1}H NMR (400 MHz, Deuterium Oxide) δ 6.78 (d, J = 4.5 Hz, 4H), 4.24 – 4.20 (m, 2H), 4.09 – 4.03 (m, 2H), 3.94 (t, J = 6.4 Hz, 1H), 3.28 – 3.18 (m, 4H), 2.89 (t, J = 7.6 Hz, 3H), 2.82 (t, J = 7.7 Hz, 2H), 1.83 (d, J = 7.9 Hz, 3H), 1.71 – 1.58 (m, 7H), 1.52 (dq, J = 15.0, 7.9 Hz, 10H), 1.24 (dd, J = 20.0, 7.2 Hz, 10H).

(EA)₂

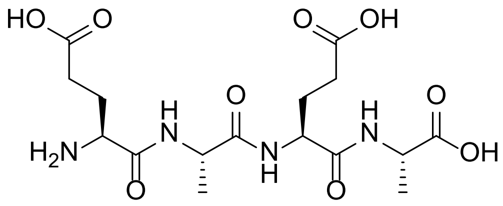

Chemical Formula: $C_{16}H_{26}N_4O_9$
Exact Mass: 418.17

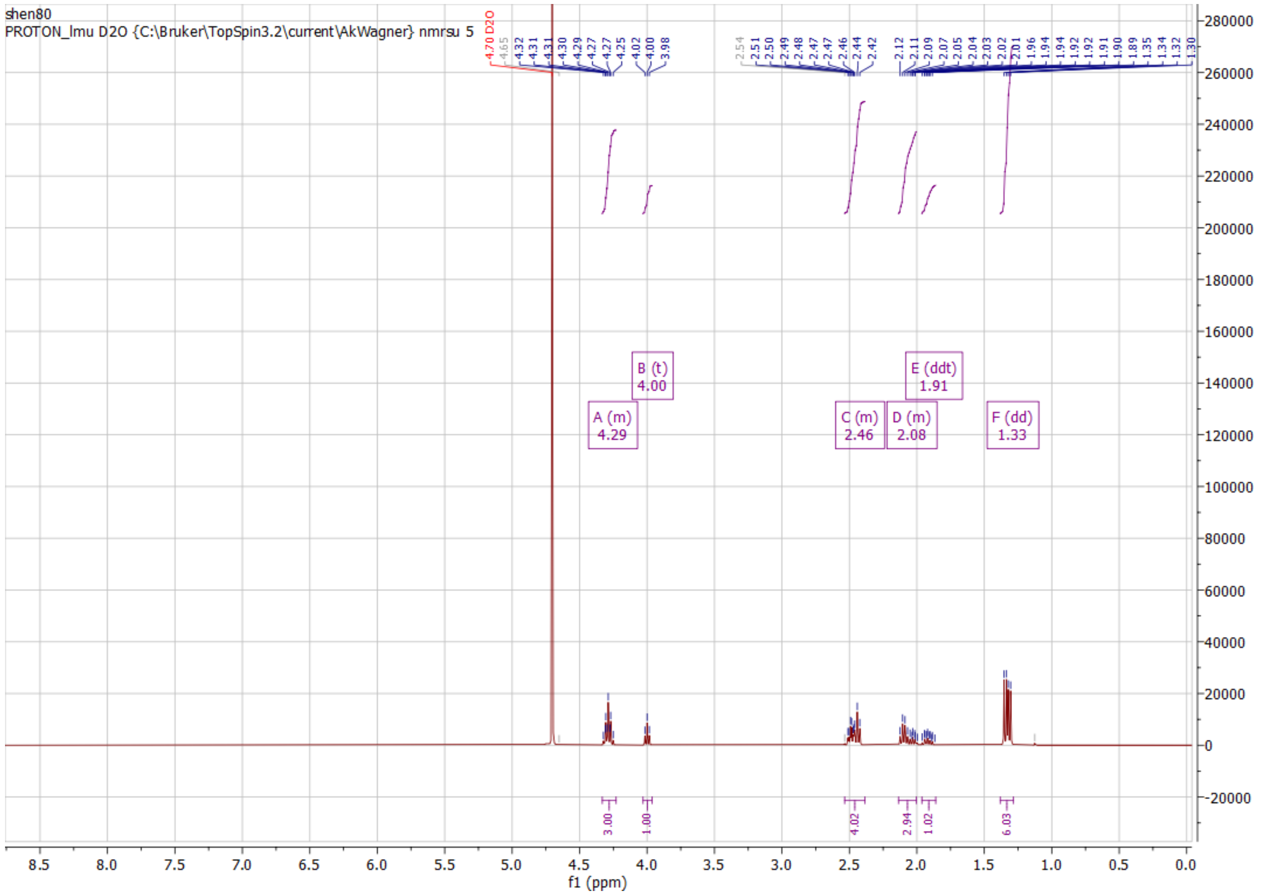

^{1}H NMR (400 MHz, Deuterium Oxide) δ 4.33 – 4.23 (m, 3H), 4.00 (t, *J* = 6.5 Hz, 1H), 2.54 – 2.38 (m, 4H), 2.14 – 2.00 (m, 3H), 1.91 (ddt, *J* = 14.3, 8.8, 7.2 Hz, 1H), 1.33 (dd, *J* = 12.2, 7.2 Hz, 6H).

(EA)₂*

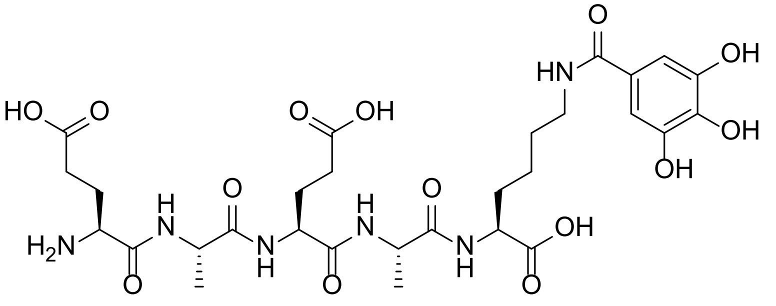

Chemical Formula: $C_{29}H_{42}N_6O_{14}$
Exact Mass: 698.28

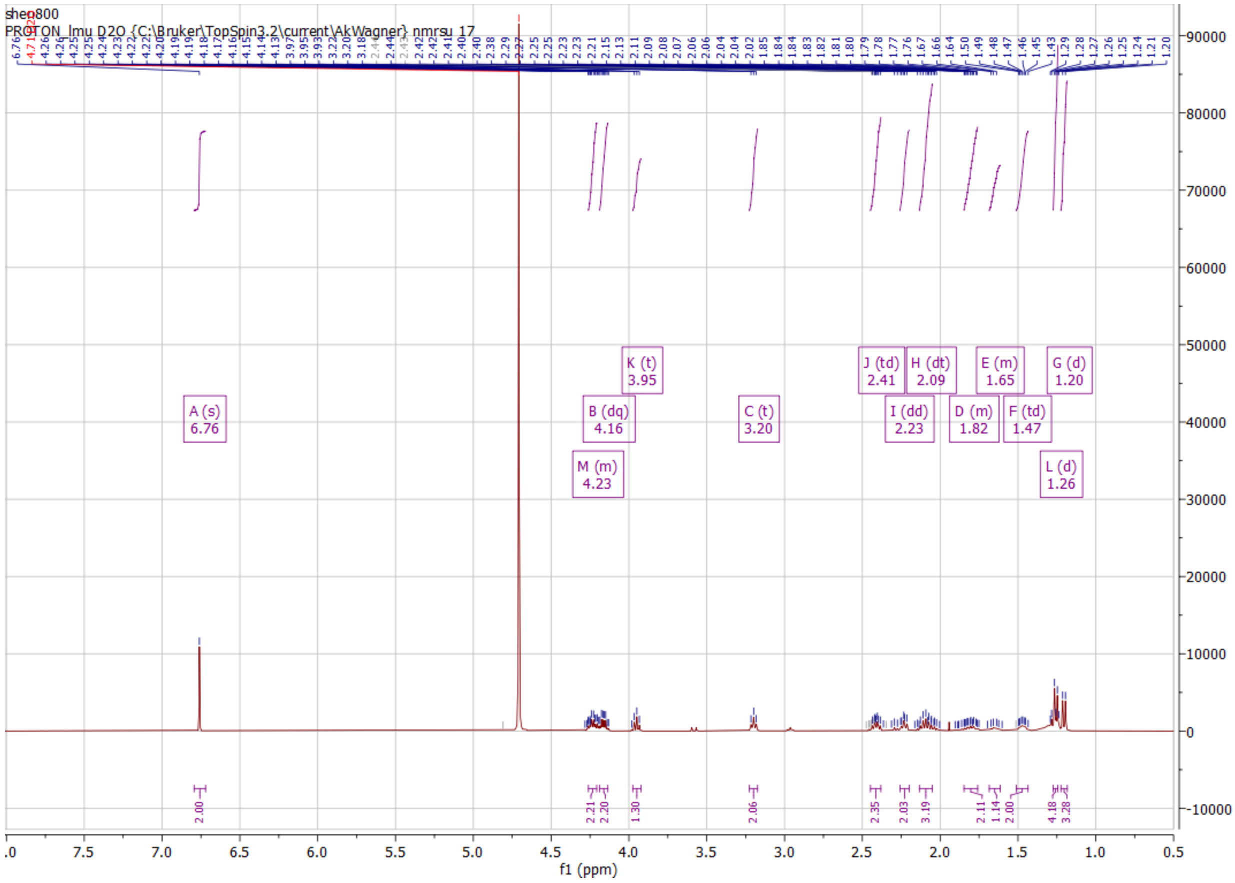

^{1}H NMR (400 MHz, Deuterium Oxide) δ 6.76 (s, 2H), 4.26 – 4.21 (m, 2H), 4.16 (dq, J = 7.3, 3.9 Hz, 2H), 3.95 (t, J = 6.5 Hz, 1H), 3.20 (t, J = 6.7 Hz, 2H), 2.41 (td, J = 7.4, 5.2 Hz, 2H), 2.23 (dd, J = 7.9, 5.9 Hz, 2H), 2.09 (dt, J = 14.3, 7.1 Hz, 3H), 1.85 – 1.76 (m, 2H), 1.68 – 1.61 (m, 1H), 1.47 (td, J = 7.6, 2.8 Hz, 2H), 1.26 (d, J = 7.3 Hz, 4H), 1.20 (d, J = 7.2 Hz, 3H).

$(EA)_2$**

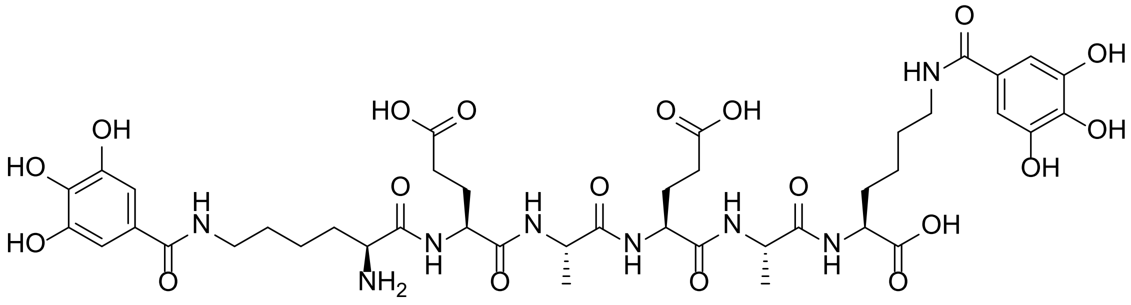

Chemical Formula: $C_{42}H_{58}N_8O_{19}$
Exact Mass: 978.38

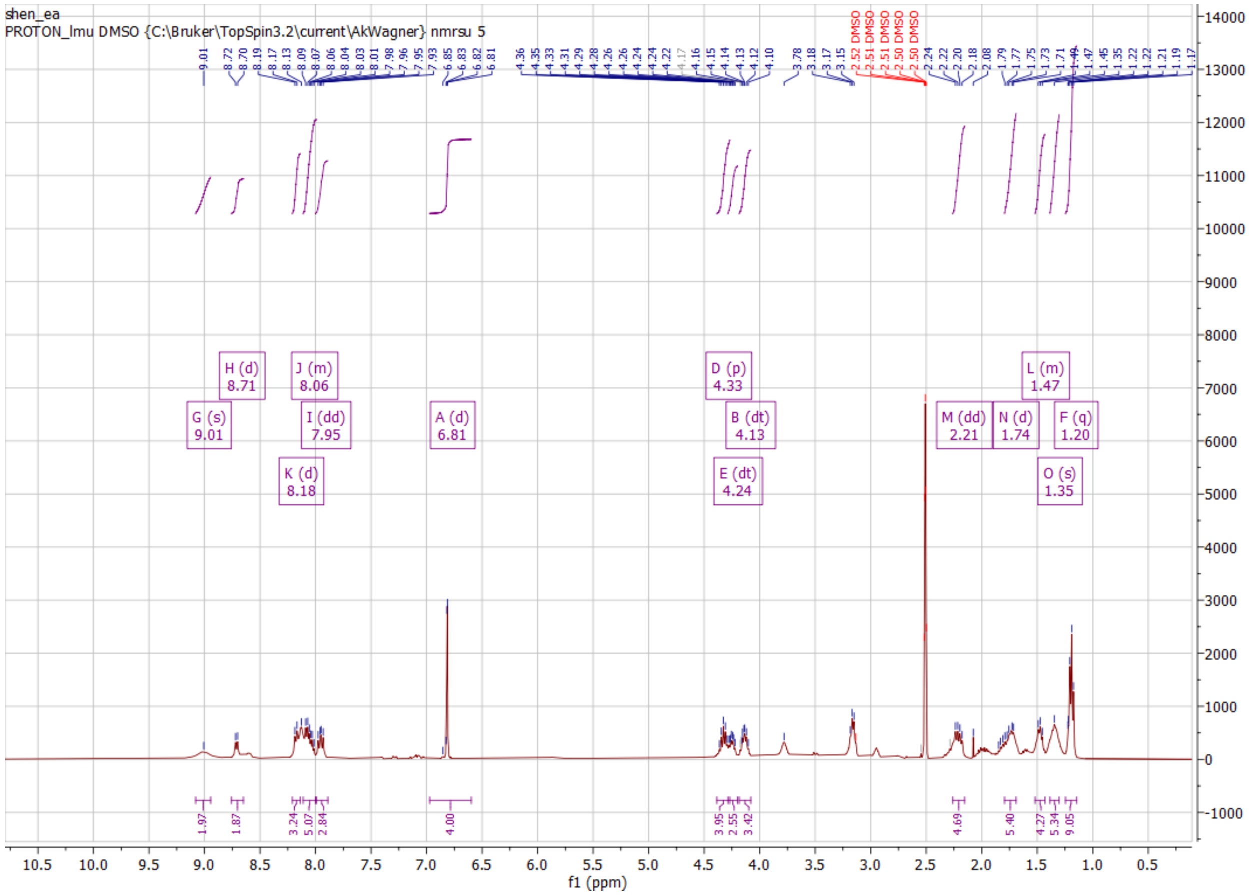

^{1}H NMR (400 MHz, DMSO-d_6) δ 9.01 (s, 2H), 8.71 (d, *J* = 7.7 Hz, 2H), 8.18 (d, *J* = 7.7 Hz, 3H), 8.11 – 7.99 (m, 5H), 7.95 (dd, *J* = 10.3, 7.5 Hz, 3H), 6.81 (d, *J* = 1.5 Hz, 4H), 4.33 (p, *J* = 7.0 Hz, 4H), 4.24 (dt, *J* = 8.0, 4.2 Hz, 3H), 4.13 (dt, *J* = 12.9, 6.0 Hz, 3H), 2.21 (dd, *J* = 16.2, 8.3 Hz, 5H), 1.74 (d, *J* = 10.9 Hz, 5H), 1.52 – 1.43 (m, 4H), 1.35 (s, 5H), 1.20 (q, *J* = 6.8 Hz, 9H).

SIO

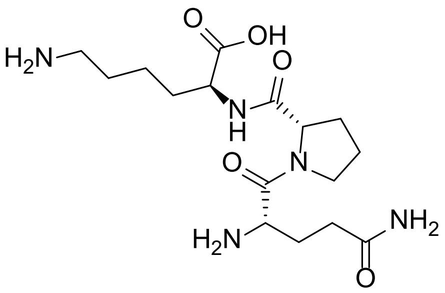

Chemical Formula: $C_{16}H_{29}N_5O_5$
Exact Mass: 371.22

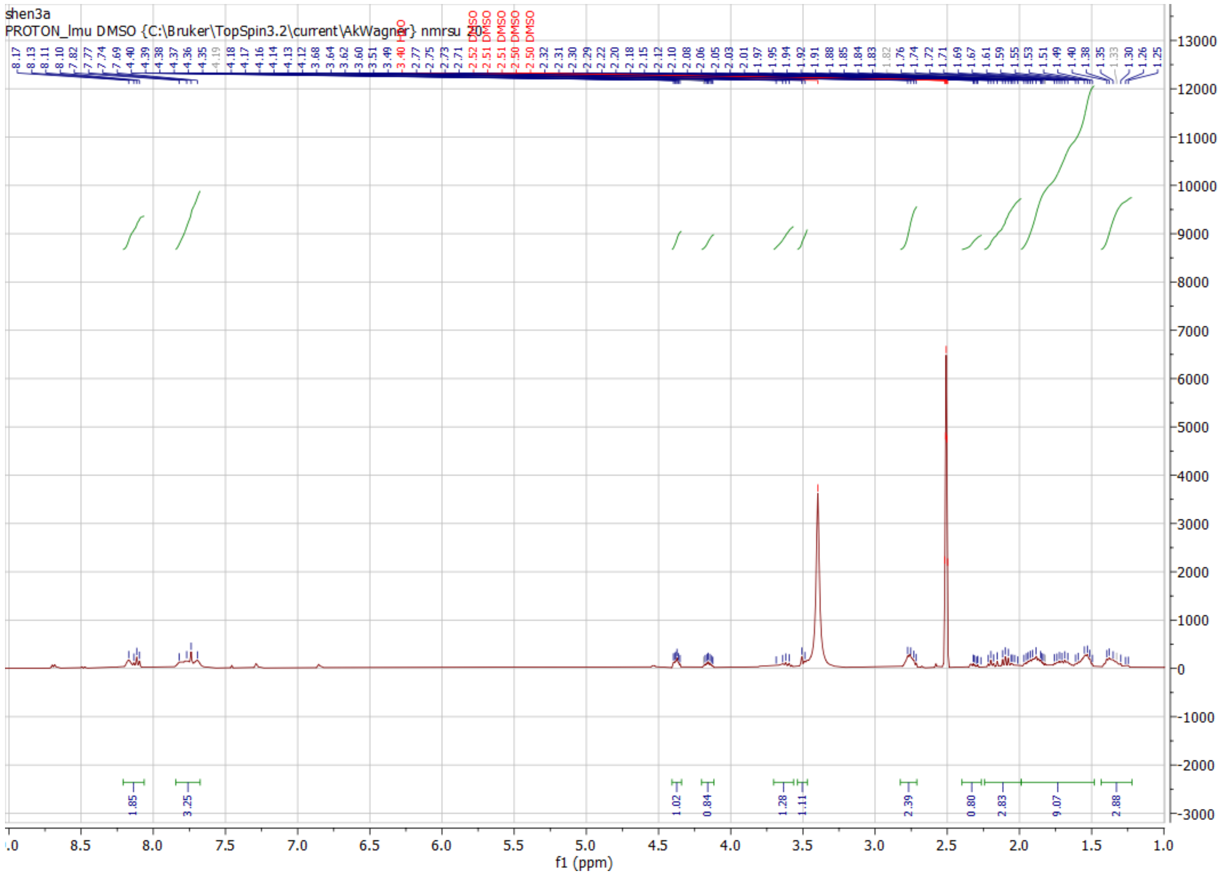

^{1}H NMR (400 MHz, DMSO-d_6) δ 8.13 (dd, *J* = 18.8, 11.2 Hz, 2H), 7.76 (q, *J* = 19.1, 17.3 Hz, 3H), 4.38 (td, *J* = 8.6, 7.6, 4.0 Hz, 1H), 4.15 (td, *J* = 8.4, 4.5 Hz, 1H), 3.70 – 3.56 (m, 1H), 3.50 (d, *J* = 8.4 Hz, 1H), 2.74 (dd, *J* = 16.3, 7.8 Hz, 2H), 2.41 – 2.24 (m, 1H), 2.19 (dd, *J* = 16.0, 8.7 Hz, 1H), 2.08 (dt, *J* = 18.1, 8.9 Hz, 2H), 1.97 – 1.81 (m, 3H), 1.71 (dt, *J* = 23.0, 7.7 Hz, 2H), 1.55 (h, *J* = 7.2 Hz, 3H), 1.44 – 1.23 (m, 3H).

SIO*

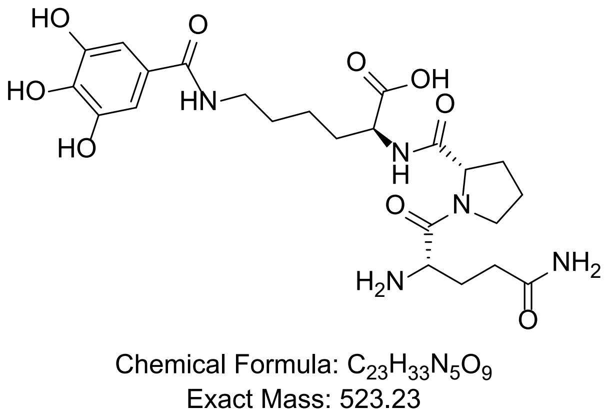

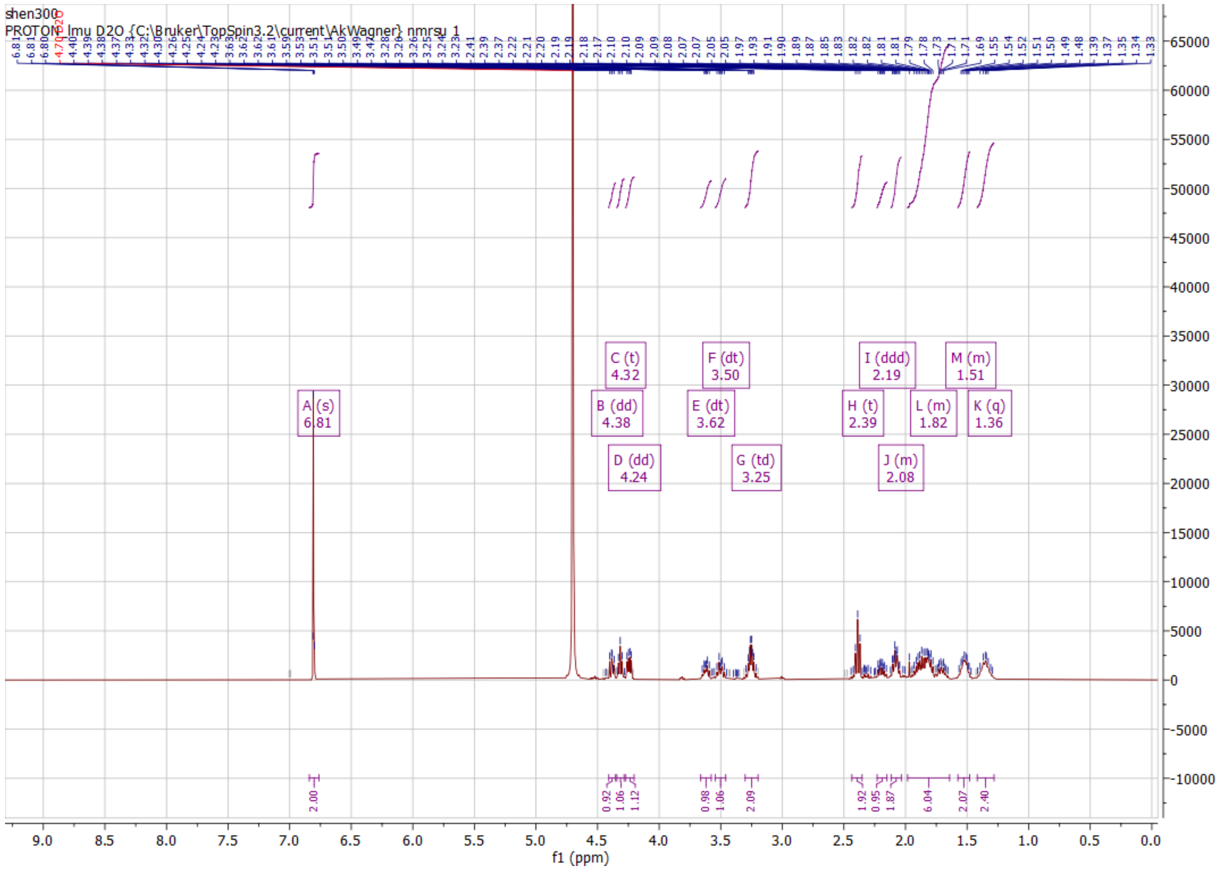

^{1}H NMR (400 MHz, Deuterium Oxide) δ 6.81 (s, 2H), 4.38 (dd, *J* = 8.4, 6.1 Hz, 1H), 4.32 (t, *J* = 5.9 Hz, 1H), 4.24 (dd, *J* = 9.1, 5.0 Hz, 1H), 3.62 (dt, *J* = 10.2, 6.4 Hz, 1H), 3.50 (dt, *J* = 10.1, 7.0 Hz, 1H), 3.25 (td, *J* = 6.7, 4.9 Hz, 2H), 2.39 (t, *J* = 7.4 Hz, 2H), 2.19 (ddd, *J* = 12.5, 8.5, 6.4 Hz, 1H), 2.12 – 2.03 (m, 2H), 1.98 – 1.64 (m, 6H), 1.57 – 1.48 (m, 2H), 1.36 (q, *J* = 9.3, 8.1 Hz, 2H).

SIO**

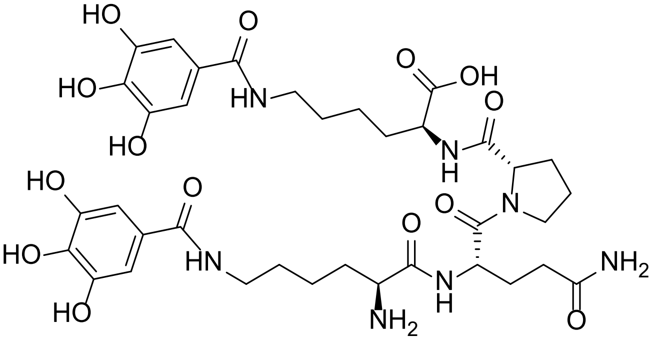

Chemical Formula: $C_{36}H_{49}N_7O_{14}$
Exact Mass: 803.33

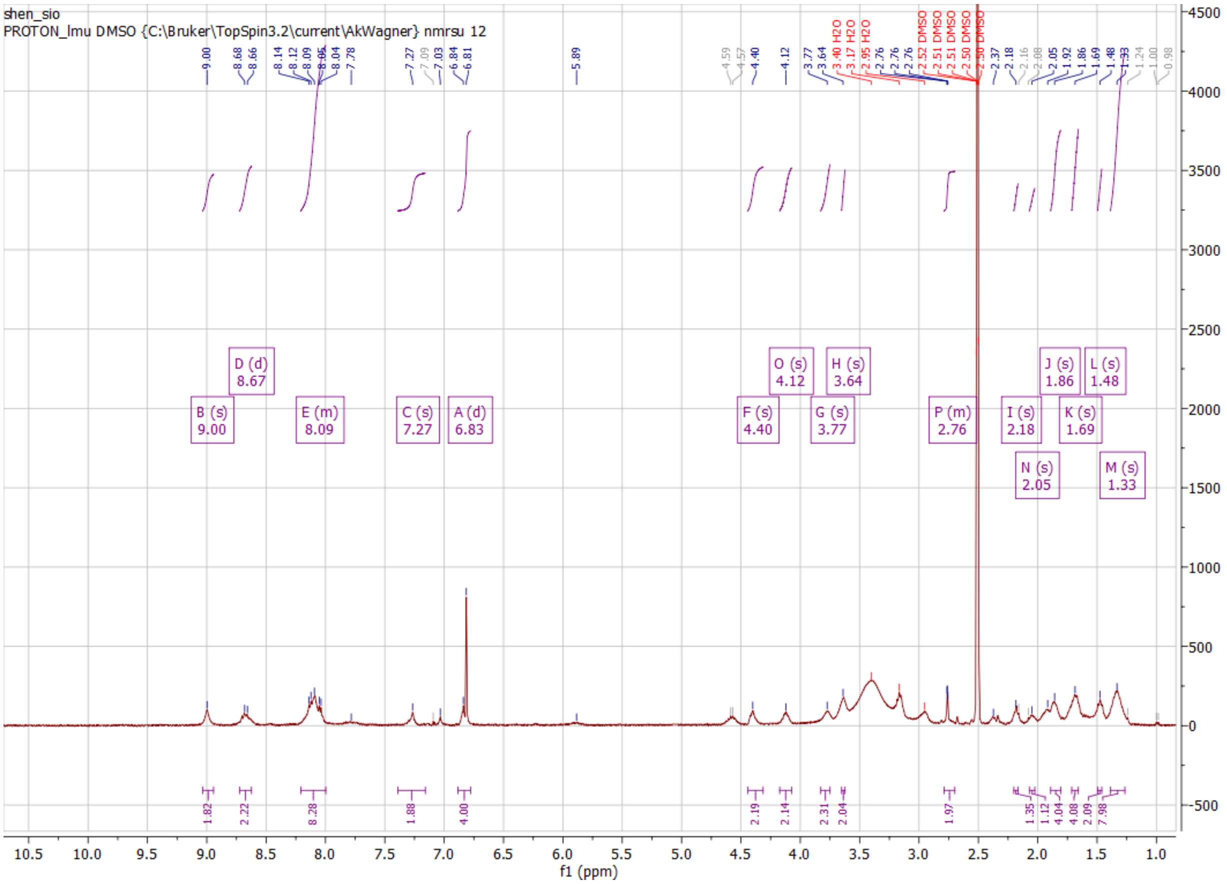

^{1}H NMR (400 MHz, DMSO-d_6) δ 9.00 (s, 2H), 8.67 (d, *J* = 9.4 Hz, 2H), 8.21 – 8.00 (m, 8H), 7.27 (s, 2H), 6.83 (d, *J* = 10.1 Hz, 4H), 4.40 (s, 2H), 4.12 (s, 2H), 3.77 (s, 2H), 3.64 (s, 2H), 2.79 – 2.70 (m, 2H), 2.18 (s, 1H), 2.05 (s, 1H), 1.86 (s, 4H), 1.69 (s, 4H), 1.48 (s, 2H), 1.33 (s, 8H).

AVP

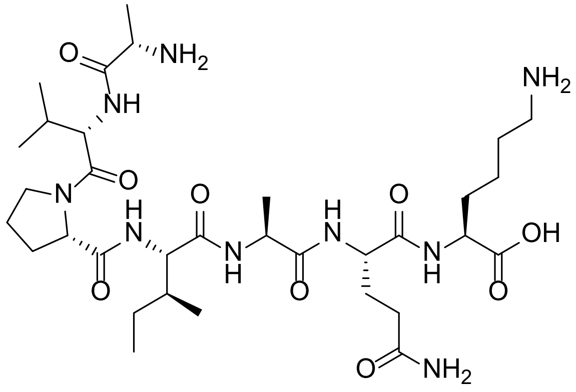

Chemical Formula: $C_{33}H_{59}N_9O_9$
Exact Mass: 725.44

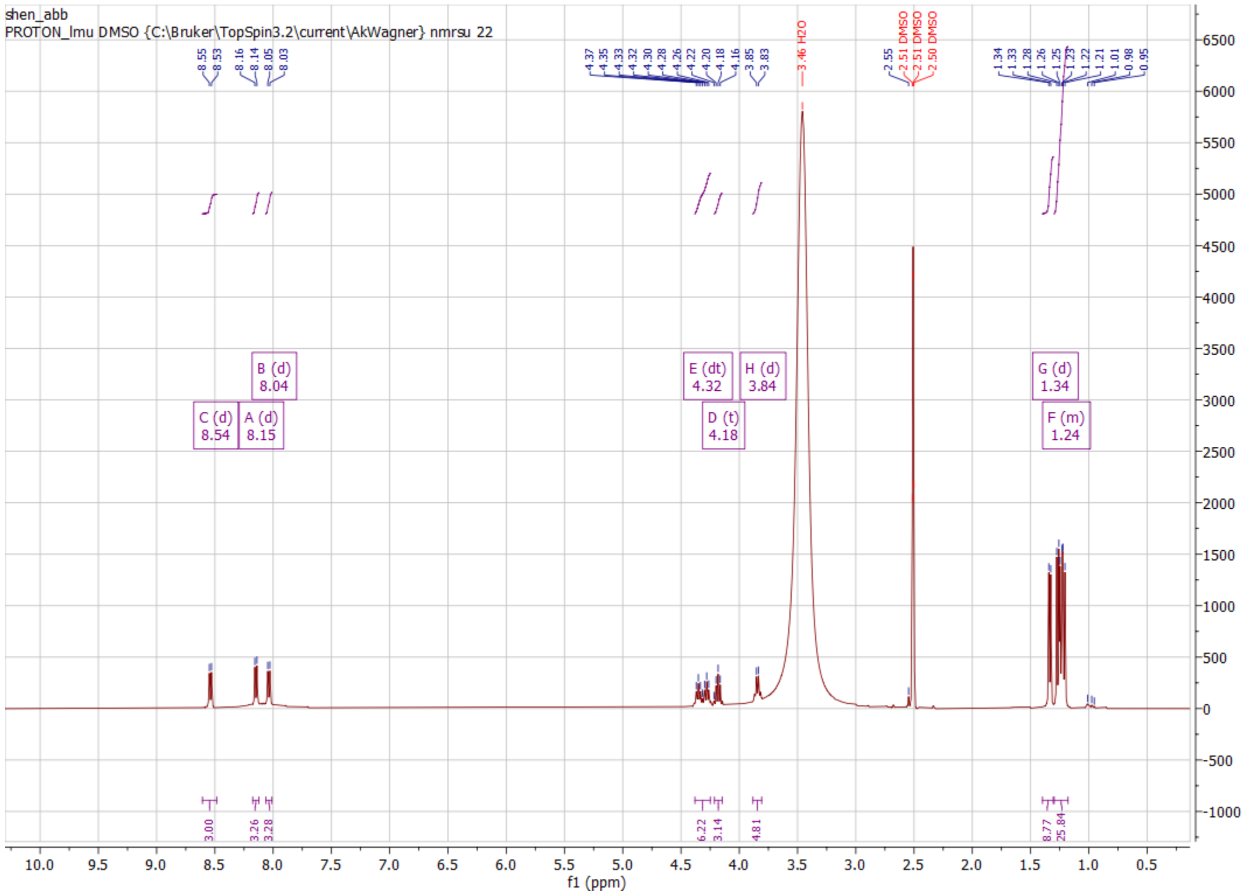

^{1}H NMR (400 MHz, DMSO-d_6) δ 8.54 (d, J = 7.5 Hz, 3H), 8.15 (d, J = 7.3 Hz, 3H), 8.04 (d, J = 7.5 Hz, 3H), 4.32 (dt, J = 28.5, 7.2 Hz, 6H), 4.18 (t, J = 7.3 Hz, 3H), 3.84 (d, J = 7.0 Hz, 5H), 1.34 (d, J = 6.9 Hz, 9H), 1.30 – 1.19 (m, 26H).

AVP*

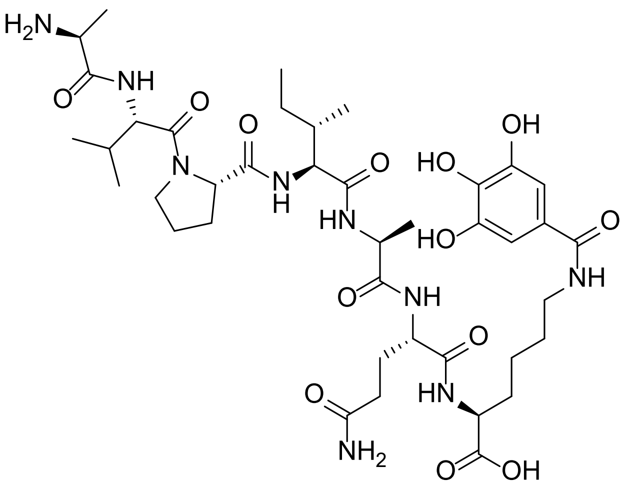

Chemical Formula: $C_{40}H_{63}N_9O_{13}$
Exact Mass: 877.45

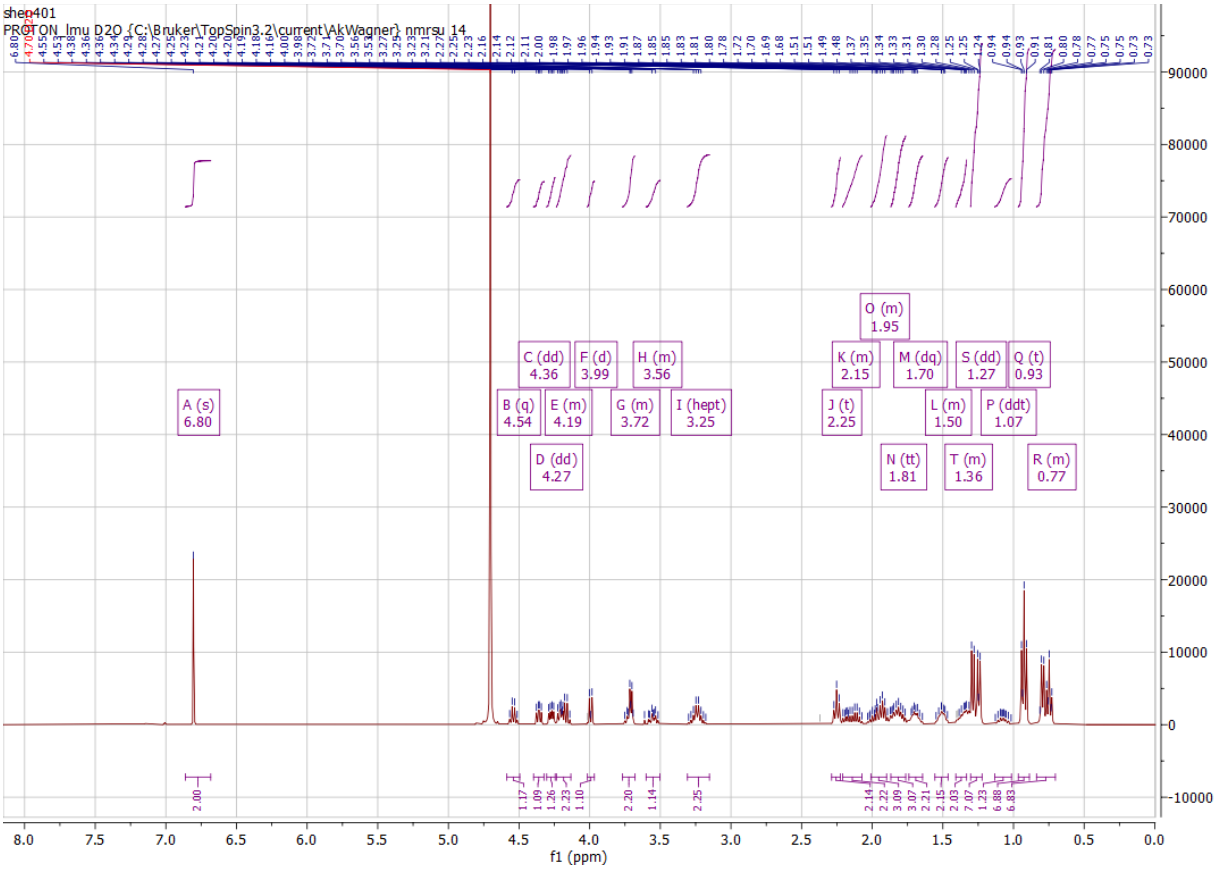

^{1}H NMR (400 MHz, Deuterium Oxide) δ 6.80 (s, 2H), 4.54 (q, *J* = 7.1 Hz, 1H), 4.36 (dd, *J* = 8.4, 5.7 Hz, 1H), 4.27 (dd, *J* = 9.3, 4.6 Hz, 1H), 4.24 – 4.13 (m, 2H), 3.99 (d, *J* = 7.7 Hz, 1H), 3.77 – 3.68 (m, 2H), 3.60 – 3.50 (m, 1H), 3.25 (hept, *J* = 6.7 Hz, 2H), 2.25 (t, *J* = 7.6 Hz, 2H), 2.21 – 2.07 (m, 2H), 2.01 – 1.90 (m, 3H), 1.81 (tt, *J* = 12.5, 6.7 Hz, 3H), 1.70 (dq, *J* = 10.8, 6.0, 4.6 Hz, 2H), 1.56 – 1.46 (m, 2H), 1.41 – 1.33 (m, 2H), 1.27 (dd, *J* = 17.2, 7.2 Hz, 7H), 1.07 (ddt, *J* = 16.3, 14.3, 7.4 Hz, 1H), 0.93 (t, *J* = 7.2 Hz, 7H), 0.84 – 0.70 (m, 7H).

AVP**

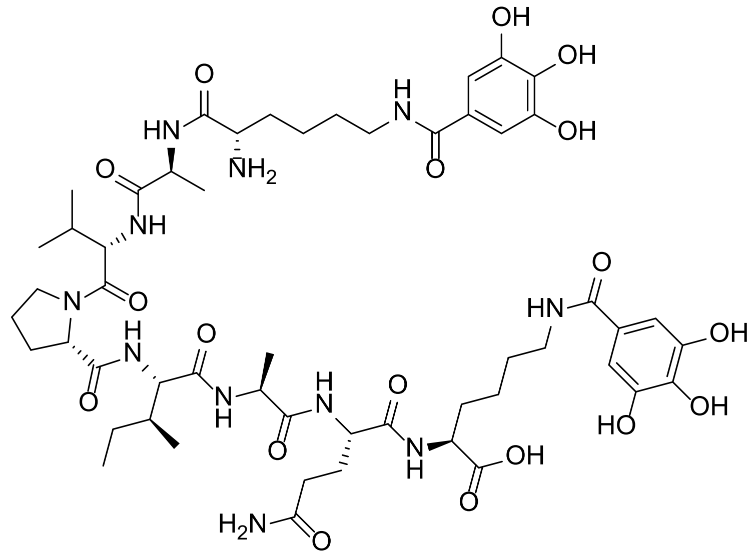

Chemical Formula: $C_{53}H_{79}N_{11}O_{18}$
Exact Mass: 1157.56

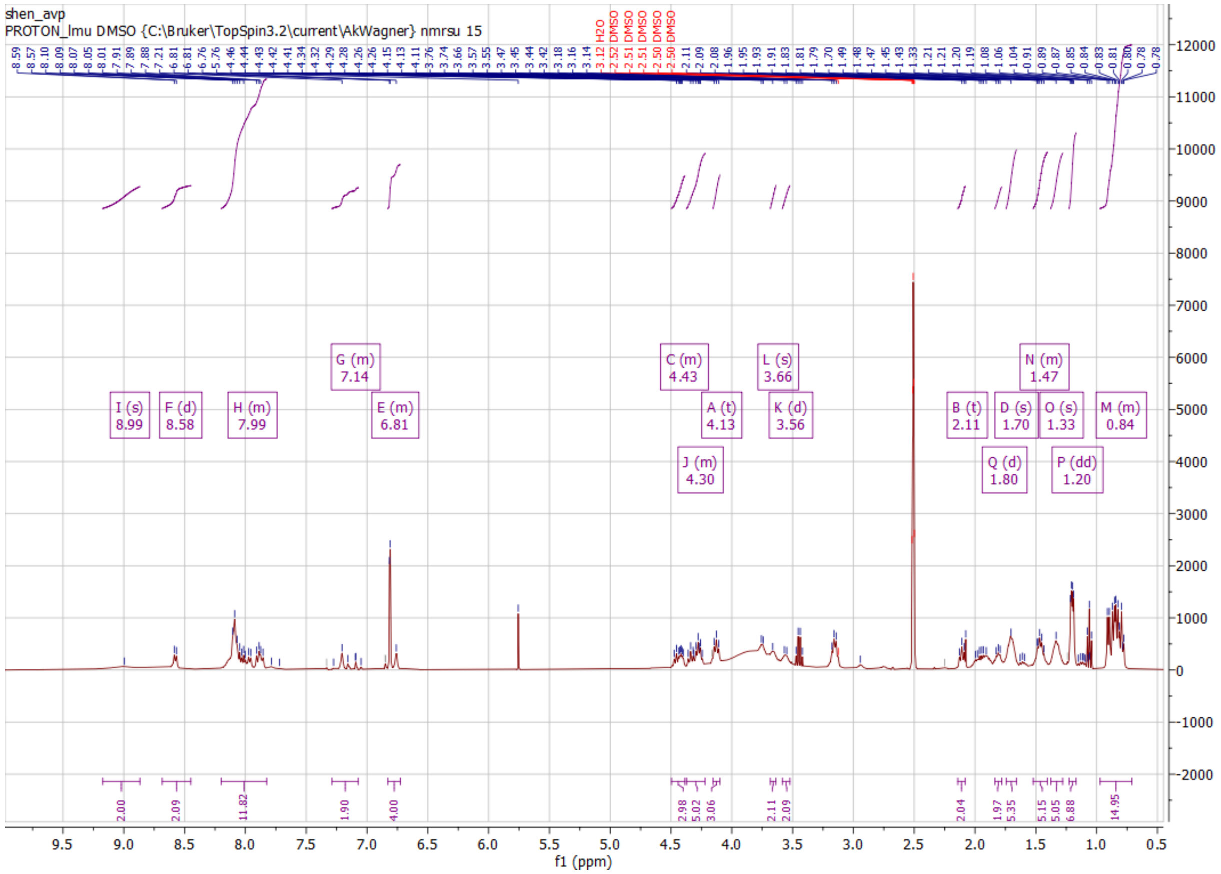

^{1}H NMR (400 MHz, DMSO-d_6) δ 8.99 (s, 2H), 8.58 (d, *J* = 7.4 Hz, 2H), 8.20 – 7.83 (m, 12H), 7.29 – 7.07 (m, 2H), 6.83 – 6.73 (m, 4H), 4.50 – 4.39 (m, 3H), 4.37 – 4.22 (m, 5H), 4.13 (t, *J* = 7.6 Hz, 3H), 3.66 (s, 2H), 3.56 (d, *J* = 7.9 Hz, 2H), 2.11 (t, *J* = 8.1 Hz, 2H), 1.80 (d, *J* = 6.2 Hz, 2H), 1.70 (s, 5H), 1.52 – 1.41 (m, 5H), 1.33 (s, 5H), 1.20 (dd, *J* = 7.1, 3.4 Hz, 7H), 0.97 – 0.71 (m, 15H).

3.3 Mass spectrometry (ESI-MS or MALDI-TOF MS)

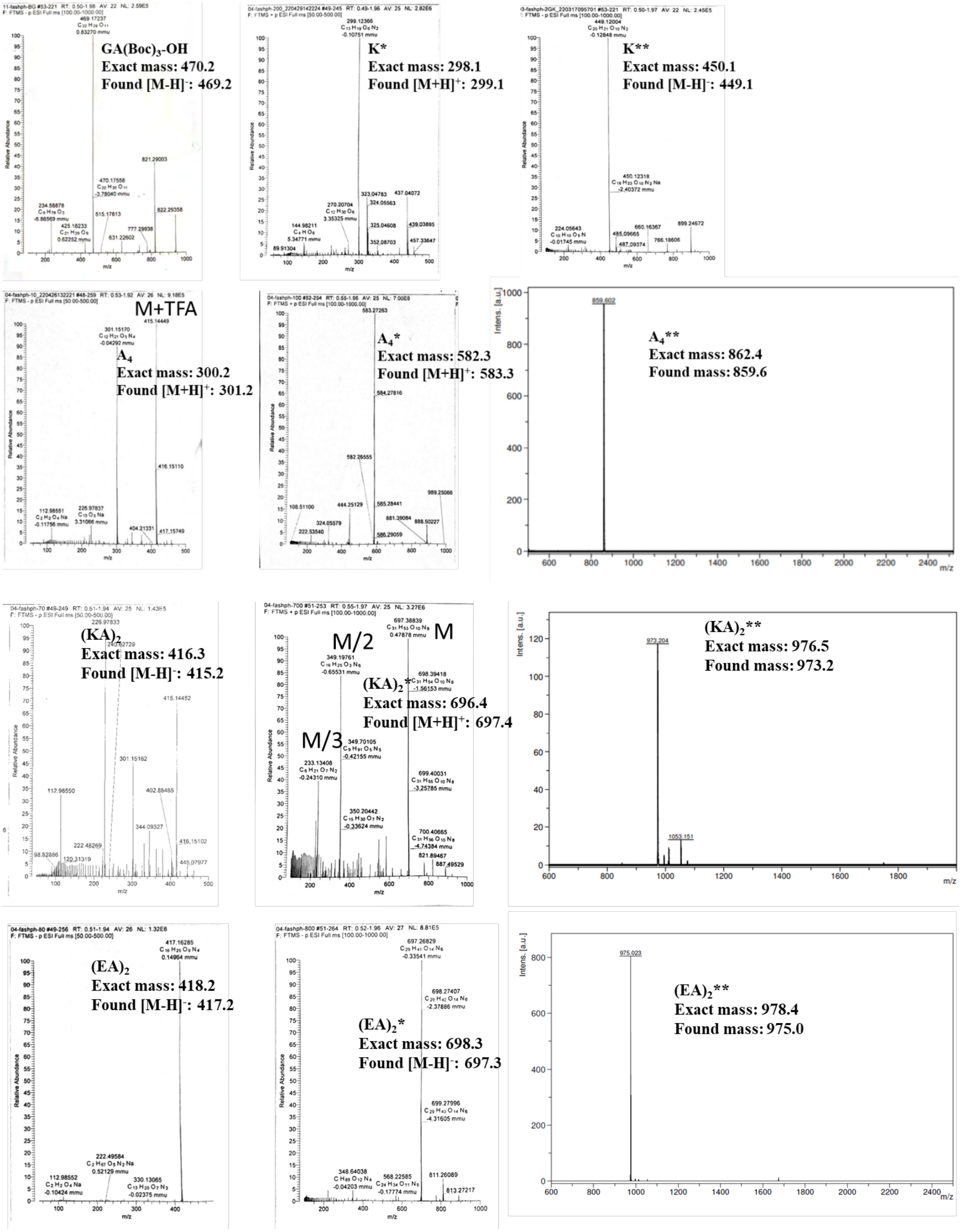
GA(Boc)3-OH
Exact mass: 470.2
Found [M-H]-: 469.2
K*
Exact mass: 298.1
Found [M+H]+: 299.1
K**
Exact mass: 450.1
Found [M-H]-: 449.1
M+TFA
A4
Exact mass: 300.2
Found [M+H]+: 301.2
A4*
Exact mass: 582.3
Found [M+H]+: 583.3
A4**
Exact mass: 862.4
Found mass: 859.6
(KA)2
Exact mass: 416.3
Found [M-H]-: 415.2
M/2
M
M/3
(KA)2*
Exact mass: 696.4
Found [M+H]+: 697.4
(KA)2**
Exact mass: 976.5
Found mass: 973.2
(EA)2
Exact mass: 418.2
Found [M-H]-: 417.2
(EA)2*
Exact mass: 698.3
Found [M-H]-: 697.3
(EA)2**
Exact mass: 978.4
Found mass: 975.0
Relative Abundance
m/z
Intens. [a.u.]

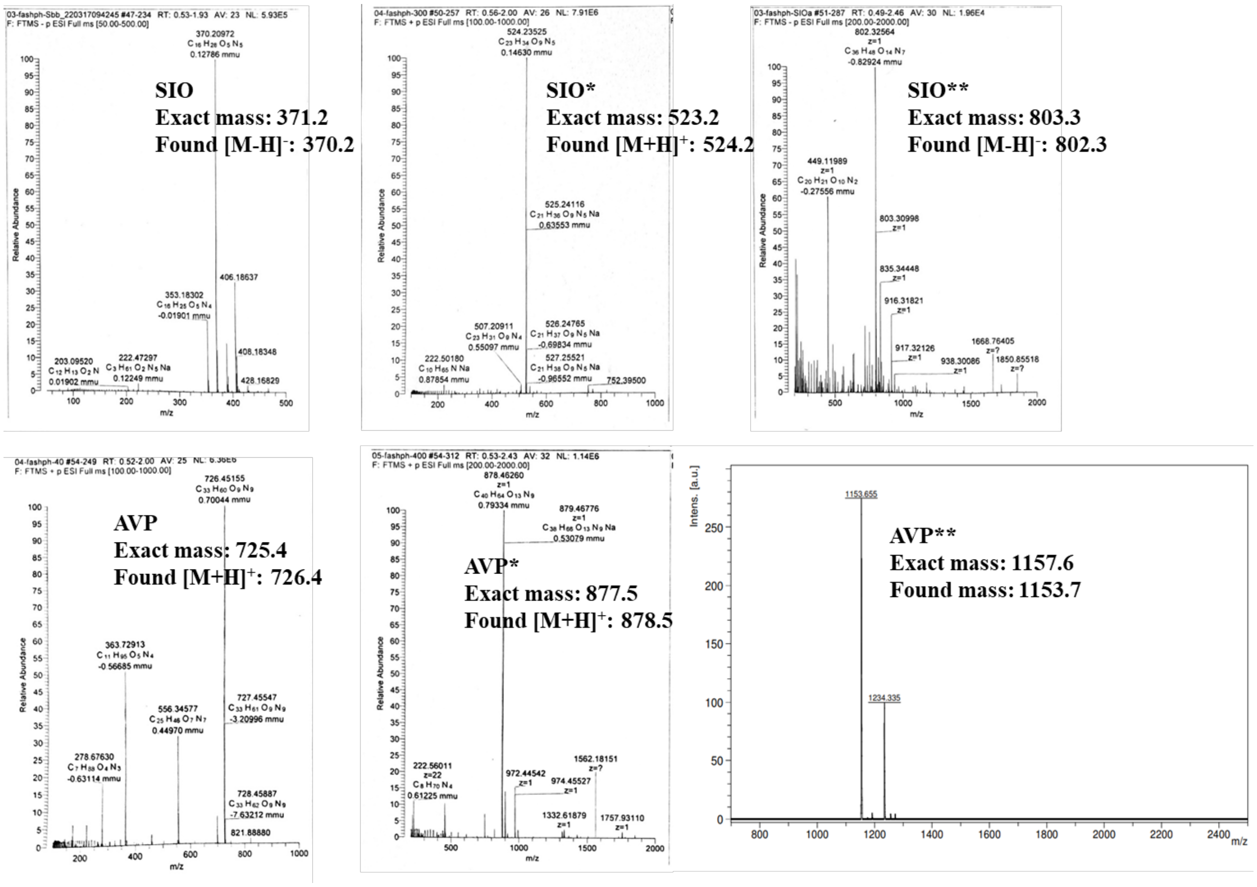

References

(1) Jin, Q.; Zhu, W.; Jiang, D.; Zhang, R.; Kutyreff, C. J.; Engle, J. W.; Huang, P.; Cai, W.; Liu, Z.; Cheng, L. Ultra-Small Iron-Gallic Acid Coordination Polymer Nanoparticles for Chelator-Free Labeling of 64Cu and Multimodal Imaging-Guided Photothermal Therapy. *Nanoscale* **2017**, *9* (34), 12609–12617. https://doi.org/10.1039/c7nr03086j.
